# Consequences of Right Heart Disease for Cardiac Electrophysiology and Arrhythmias: Cellular and Structural Mechanisms

**DOI:** 10.1016/j.cjco.2025.06.013

**Published:** 2025-06-23

**Authors:** Orlane Neuilly, Ngoc Anh Lisa Le, Paul Khairy, Roddy Hiram

**Affiliations:** a Montreal Heart Institute, Faculty of Medicine, University of Montreal, Montreal, Quebec, Canada

**Keywords:** Right heart disease, inflammation, atrial fibrosis, cardiac arrhythmias, atrial fibrillation

## Abstract

Conditions provoking the electrical and structural-functional remodelling of the myocardium can lead to the development of heart rhythm disorders, including atrial fibrillation (AF) and ventricular tachyarrhythmias that can cause sudden cardiac death. Right heart disease (RHD) causes progressive structural and functional remodelling of the right heart responsible for right ventricular and atrial dysfunction and arrhyhmias. Conditions contributing to the development of RHD include left heart disease, pulmonary arterial hypertension, congenital heart disease, right-sided myocardial infarction due to coronary artery occlusion, and amyloidosis. In adult patients with RHD associated with pulmonary arterial hypertension, the prevalence of AF is about 20%, and in adult patients with arrhythmogenic right ventricular cardiomyopathy, it is 14%. A study has suggested that compared to non patients without congenital heart disease, AF appears 30 years earlier in adult patients with congenital heart disease, with a 10-20-fold-higher incidence. This narrative review article aims to review knowledge about the pathophysiology of RHD associated with cardiac arrhythmia. Evidence is reported about the mechanisms underlying the initiation and maintenance of the arrhythmogenic substrate in RHD. We summarize the available experimental approaches to study RHD associated with cardiac arrhythmia, including *in vitro* models (isolated cardiomyocytes, fibroblasts) and *in vivo* models (monocrotaline, pulmonary artery banding, Sugen/hypoxia). In addition, we discuss potential future strategies targeting myocardial inflammation and fibrosis in the prevention of cardiac arrhythmia in RHD.

## Résumé

Les affections qui provoquent un remodelage électrique, fonctionnel et structurel du myocarde peuvent entraîner l’apparition de troubles du rythme cardiaque, comme la fibrillation auriculaire (FA) et les tachyarythmies ventriculaires, pouvant elles-mêmes entraîner une mort subite d’origine cardiaque. La cardiopathie droite cause un remodelage fonctionnel et structurel graduel du cœur droit, à l’origine de dysfonctions ventriculaires et auriculaires droites et d’arythmies. Les affections contribuant à l’apparition d’une cardiopathie droite sont les cardiopathies gauches, l’hypertension artérielle pulmonaire, les cardiopathies congénitales, l’infarctus du myocarde du côté droit attribuable à une occlusion de l’artère coronaire et l’amylose. Chez les adultes atteints d’une cardiopathie droite associée à l’hypertension artérielle pulmonaire, la prévalence de la FA est d’environ 20 %; elle est de 14 % chez les adultes atteints d’une cardiomyopathie arythmogène du ventricule droit. Une étude indique que la FA apparaît 30 ans plus tôt chez les adultes atteints d’une cardiopathie congénitale, et que son incidence est de 10 à 20 fois plus élevée, que chez ceux qui n’en sont pas atteints. Cet article de synthèse vise à faire le point sur les connaissances relatives à la physiopathologie de la cardiopathie droite associée aux arythmies cardiaques. Les données probantes relatives aux mécanismes qui sous-tendent la formation et le maintien du substrat arythmogène associé à la cardiopathie droite y sont présentées. Nous résumons les approches expérimentales disponibles pour étudier la cardiopathie droite associée aux arythmies cardiaques, notamment les modèles *in vitro* (cardiomyocytes isolés, fibroblastes) et *in vivo* (monocrotaline, bandage de l’artère pulmonaire, sugen/hypoxie). Nous présentons également les stratégies ciblant l’inflammation et la fibrose myocardiques qu’il est possible de mettre en place à l’avenir pour prévenir les arythmies cardiaques en cas de cardiopathie droite.

Right-heart disease (RHD), characterized by right-sided cardiac remodelling and dysfunction affecting the right ventricle, the tricuspid valve, and the right atrium, is among the important conditions causing cardiac rhythm disorders.^1^ In RHD, the right ventricle and right atrium are subjected to pressure and/or volume overload due to abnormalities in pulmonary vascular function, valvular integrity, and cardiac structure.^1^^,^^2^

Contributing conditions, including left-heart disease, characterized by left-sided cardiac failure, can lead to increased pulmonary venous pressure, and secondarily, arterial pressure.^3^ Left-heart disease-induced pulmonary hypertension exposes the right ventricle to pressure overload, causing right-ventricular (RV) hypertrophy, and if sustained, RV failure.^4^ Lung diseases such as chronic obstructive pulmonary disease, cystic fibrosis, repeated pulmonary embolism, pulmonary hypertension, and pulmonary arterial hypertension, are responsible for increased blood arterial pressure, potentially leading to RV hypertrophy and RV failure.^5^, ^6^, ^7^, ^8^ Valvopathies, including pulmonary and tricuspid valve stenosis and regurgitation, also can cause RHD due to RV volume and pressure overload.^9^ In addition, congenital heart disease is an important cause of RHD via pressure and/or volume overload to the right ventricle (eg, atrial or ventricular septal defects with shunts, RV outflow tract obstruction, Ebstein anomaly, tetralogy of Fallot, transposition of the great arteries, and single right ventricle).^10^

**Figure 1:**
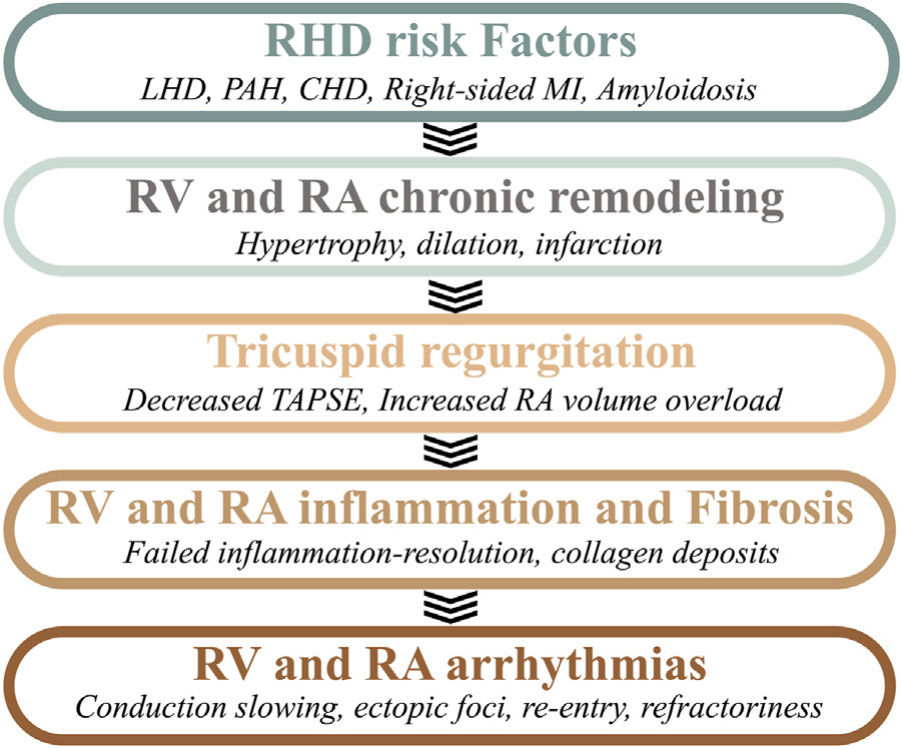
Proposed mechanical cascade from right heart disease (RHD) to cardiac arrhythmias. Conditions such as left heart disease (LHD), pulmonary artery hypertension (PAH), congenital heart disease (CHD), myocardial infarction (MI), or amyloidosis can provoke right heart disease (RHD), identified by chronic right ventricular (RV) and right atrial (RA) remodelling characterized by myocardial hypertrophy and dilation. Disturbance of RV and RA structure is accompanied by abnormal tricuspid valve closure. In echocardiography, decreased tricuspid annulus plane systolic excursion (TAPSE) is an important sign of RHD. During RHD, the RV and RA myocardium undergo activation and maintenance of inflammation due to the perpetuation of inflammatory signals as the disease aggravates. Chronic inflammation often stimulates collagen deposits, contributing to the development of fibrosis. The presence of fibrosis is recognized to impair cardiac electrical conduction, promoting reentry and arrhythmias in the right ventricle and right atrium.

An important consequence of RHD is the development of chronic structural remodelling affecting the right ventricle and right atrial (RA) myocardium.^11^, ^12^, ^13^ Clinical evidence suggests that patients with RHD have an increased risk of developing cardiac arrhythmias.^11^ Of these, atrial fibrillation (AF) is the most common, affecting 20%-25% of patients with pulmonary hypertension,^12^^,^^13^ and ventricular tachycardia and fibrillation are the most dangerous and life-threatening, occurring most commonly in cases of severe RHD and right-heart failure (RHF).^14^ The diagnosis and management of RHD—aiming to optimize RV function, control conduction abnormalities, and promote normal rhythm—are major clinical concerns^13^^,^^15^^,^^16^ (Fig. 1).

In this narrative review paper, we aim to discuss the following: (i) the conditions and pathophysiological events that might be responsible for the development of cardiac arrhythmias in RHD; (ii) the specific cellular and structural mechanisms underlying the vulnerability of RHD patients to cardiac arrhythmias; (iii) innovative experimental approaches contributing to a better understanding of arrhythmogenesis in RHD; (iv) specific therapeutic approaches currently available to manage cardiac arrhythmias in RHD patients; and (v) potential future strategies to prevent and treat arrhythmias in RHD patients.

## Methods

Research reports and previous review articles were identified as related to RHD and cardiac rhythm disorders, including AF and ventricular tachyarrhythmia and/or ventricular fibrillation, in PubMed, MEDLINE, ScienceDirect, and Scopus, from their inception until January 2025. The following search formula was used: (“right heart disease” OR “right heart failure” OR “right-sided cardiac disease”) [title/abstract] + (“heart”, “cardiac”) [title/abstract] + (“atrial fibrillation”), “tetralogy” AND “action potential”, “pulmonary hypertension” AND “action potential”, and “right ventricle” “disease” AND “action potential”. Additional articles were identified based on citations in the papers cited. Only articles with English full texts were reviewed.

## Conditions Contributing to the Development of RHD Associated with Cardiac Arrhythmias

### RHD induced by left-ventricular (LV) failure

Anatomically, the right ventricle and left ventricle share the interventricular septum.^17^ The shape and position of the septum can be affected by changes in pressure and volume occurring in one ventricle or the other.^18^ Hence, the function of one ventricle can influence the function of the other.^17^^,^^18^ This ventricular interdependence plays an essential role in the pathophysiology of LV and RV dysfunctions.^19^ In this context, left-ventricular failure can induce RHD due to the interconnected nature of the heart’s structure and circulatory system.^3^ Elevated pressures and dysfunction in the left heart can lead to a cascade of changes in the right heart.^3^

When left-heart failure is accompanied by reduced ejection fraction (HFrEF), the homeostatic responses to the decreased cardiac output involve the activation of the sympathetic nervous system, the renin-angiotensin-aldosterone system, and the arginine vasopressin system.^20^^,^^21^ Prolongation of such neurohormonal activation promotes systemic vasoconstriction and contributes to left-heart failure-associated pressure and/or volume overload and tachyarrhythmias affecting the left and right sides of the heart.^20^^,^^21^

Substantial sex differences in left HFrEF have an impact on every aspect of the disease, including the potential repercussions on RHD development.^22^ A study has suggested that the lifetime risk of HFrEF is higher in men (10.6%) than it is in women (5.8%).^23^ Men tend to develop HFrEF at a younger age, whereas women with HFrEF are generally older and have more severe complications and worse quality of life.^24^

**Figure 2:**
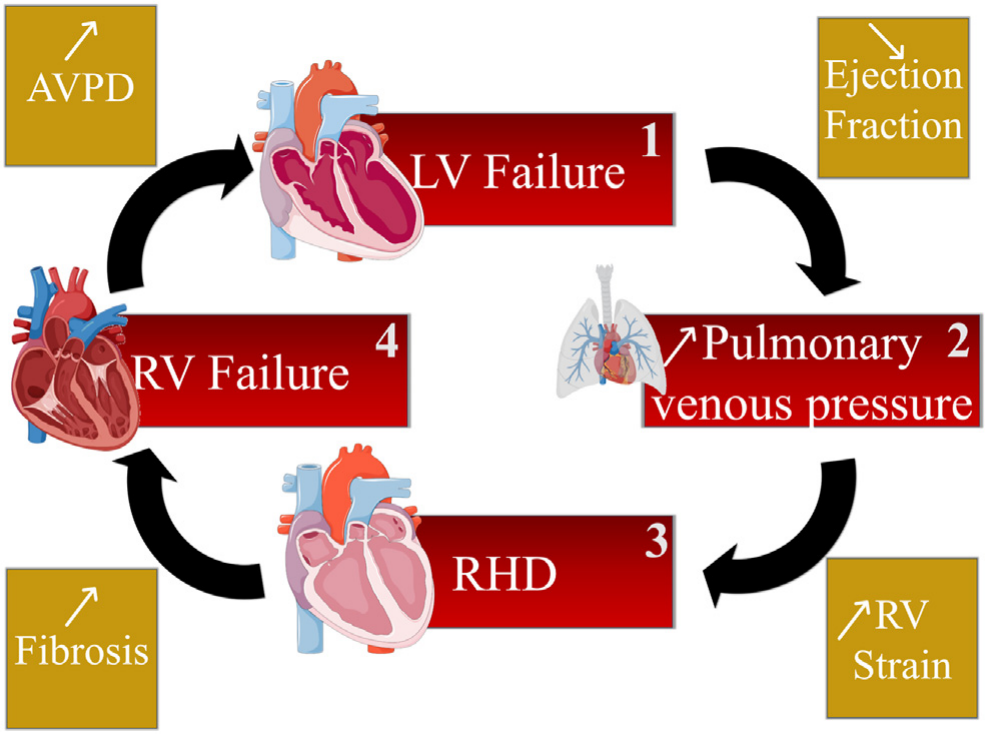
Cardiac and pulmonary remodeling from left heart disease to right heart disease (RHD). Left ventricular (LV) failure can lead to reduced ejection fraction, which provokes an elevation of volume in the lung, characterized by increased pulmonary venous pressure. Subsequently, abnormally elevated pulmonary pressure enhances the right ventricular (RV) strain, inducing RHD identified by RV hypertrophy and dilation, tricuspid valve dysfunction, tricuspid regurgitation, and right atrial thickening and enlargement. Prolonged RHD can be accompanied with the apparition of RV and right atrial fibrosis, ultimately leading to RV failure. As the 4 cardiac cavities are interconnected, aggravation of RHD and right heart failure can affect the left side via atrioventricular plane displacement (AVPD).

In HFrEF, volumes of blood can back up into the left atrium and subsequently into the pulmonary veins.^25^ Chronic elevation of pulmonary venous pressure due to LV failure leads to conditions such as pulmonary congestion (edema) and pulmonary arterial hypertension.^25^^,^^26^ The left heart disease-induced elevated pulmonary venous pressure creates higher resistance for blood flowing from the right ventricle into the pulmonary artery, causing RV pressure and/or volume overload.^3^^,^^13^^,^^27^ As the right ventricle pumps against a higher pressure in the lungs, the right ventricle’s myocardium progressively hypertrophies and the chamber dilates until loss of contractile function, tricuspid valve regurgitation, and right atrial dilation occur, altogether contributing to RHD^27^ (Fig. 2).

### RHD induced by pressure and volume overload due to lung disease

When the lung is subjected to conditions associated with decreased pulmonary circulation and increased blood pressure, the right ventricle can be affected, leading to RHD.^28^ In conditions such as pulmonary hypertension, chronic obstructive pulmonary disease, acute respiratory distress syndrome, and pulmonary embolism, the narrowing, stiffening, or obstruction of the small pulmonary arteries contributes to an increase in the resistance to blood circulation, creating an important elevation of RV afterload.^5^^,^^7^^,^^13^^,^^29^ Over time, this increased pressure leads to RV hypertrophy and, eventually, RHF.^30^^,^^31^

Studies have shown that women are more likely to develop pulmonary hypertension than are men, but women exhibit better RV adaptation to chronic pressure overload and superior RV function, compared to those in men.^32^ In contrast, men tend to have a lower RV ejection fraction and are more susceptible to RV dysfunction and failure in RHD.^33^

**Figure 3:**
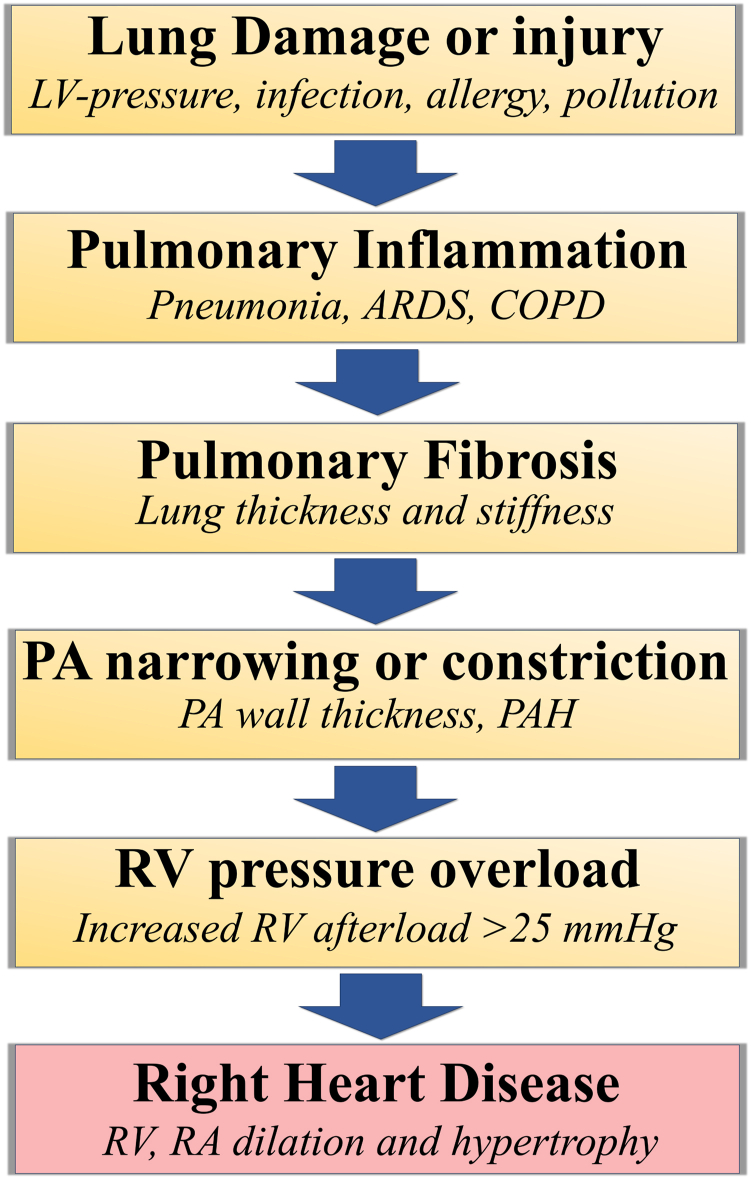
Pulmonary obstructive disorders are important contributing conditions for right heart disease. Various conditions, including left heart disease, infections, allergies, or pollution, can provoke lung damage and injuries, leading to the initiation of pulmonary inflammation. Lung conditions such as pneumonia, acute respiratory distress syndrome (ARDS), and chronic obstructive pulmonary disease have, in common, an important inflammatory status. Unresolved pulmonary inflammation leads to pulmonary fibrosis, characterized by lung thickness and stiffness. The pulmonary vessels and arteries are sensitive to pulmonary inflammation. Conditions promoting thickening of the pulmonary artery (PA) wall are characterized by increased PA pressure. PA hypertension (PAH) is a specific and complex disorder in which the PA pressure exceeds 25 mm Hg, provoking elevated right ventricular (RV) workload. Over time, lung disease can affect the right ventricle and right atrium (RA) by provoking hypertrophy and dilation. COPD, chronic obstructive pulmonary disorder; LV, left ventricular.

Some conditions in which pulmonary blood flow is limited by RV outflow tract obstruction are present from the time of fetal development and birth, such as congenital heart disease, in which the normal functioning of the valves (including the pulmonary valve and tricuspid valve) and other parts of the circulatory system are affected, leading to RHD.^34^^,^^35^ Dysfunction of the pulmonary valve and tricuspid valve may result in complications such as RV and RA hypertrophy, dilation, and RHF.^36^^,^^37^ Tricuspid valve dysfunction affects the flow of blood from the right atrium to the right ventricle.^37^^,^^38^ When the tricuspid valve does not close efficiently, tricuspid regurgitation occurs, characterized by the fact that volumes of blood can flow backward into the right atrium, leading to volume overload in the right atrium and subsequent RA dilation.^38^ Pulmonary valve dysfunction or stenosis disrupts the flow of blood from the right ventricle to the pulmonary artery trunk, increasing the workload on the right side of the heart.^39^ Progressively, the prolonged pressure overload can lead to RV hypertrophy, dilation, and RHD^39^, ^40^, ^41^, ^42^, ^43^ (Fig. 3).

### RHD induced by local RV and RA dysfunctions: infarction and amyloidosis

Although less frequent than the risk factors described above, RHD can be provoked by conditions affecting the right ventricle and the right atrium locally. The right side of the heart receives blood from the right coronary artery and the left anterior descending artery.^44^ The right coronary artery supplies blood mainly to the right ventricle and right atrium.^44^, ^45^, ^46^ The right coronary artery branches into the right marginal artery, also known as the acute marginal artery, which irrigates the right ventricle’s anterior and lateral walls, and the posterior descending artery in about 80% of patients.^47^ In patients with left circumflex artery dominance (5% to 10% of patients), the posterior descending artery originates from the left circumflex artery.^48^ About 10%-20% of patients have co-dominance, in which both the right coronary artery and the left circumflex artery supply the posterior descending artery.^47^^,^^48^ The posterior descending artery irrigates the RV inferior wall and the posterior portion of the interventricular septum.^44^^,^^45^ The left anterior descending artery perfuses the left ventricle, as well as the anterior portion of the interventricular septum and the inferior portion of the right ventricle.^45^ Understanding the blood supply to the right ventricle is crucial for the diagnosis and management of RHD, including RV infarction.^44^, ^45^, ^46^, ^47^, ^48^

**Figure 4:**
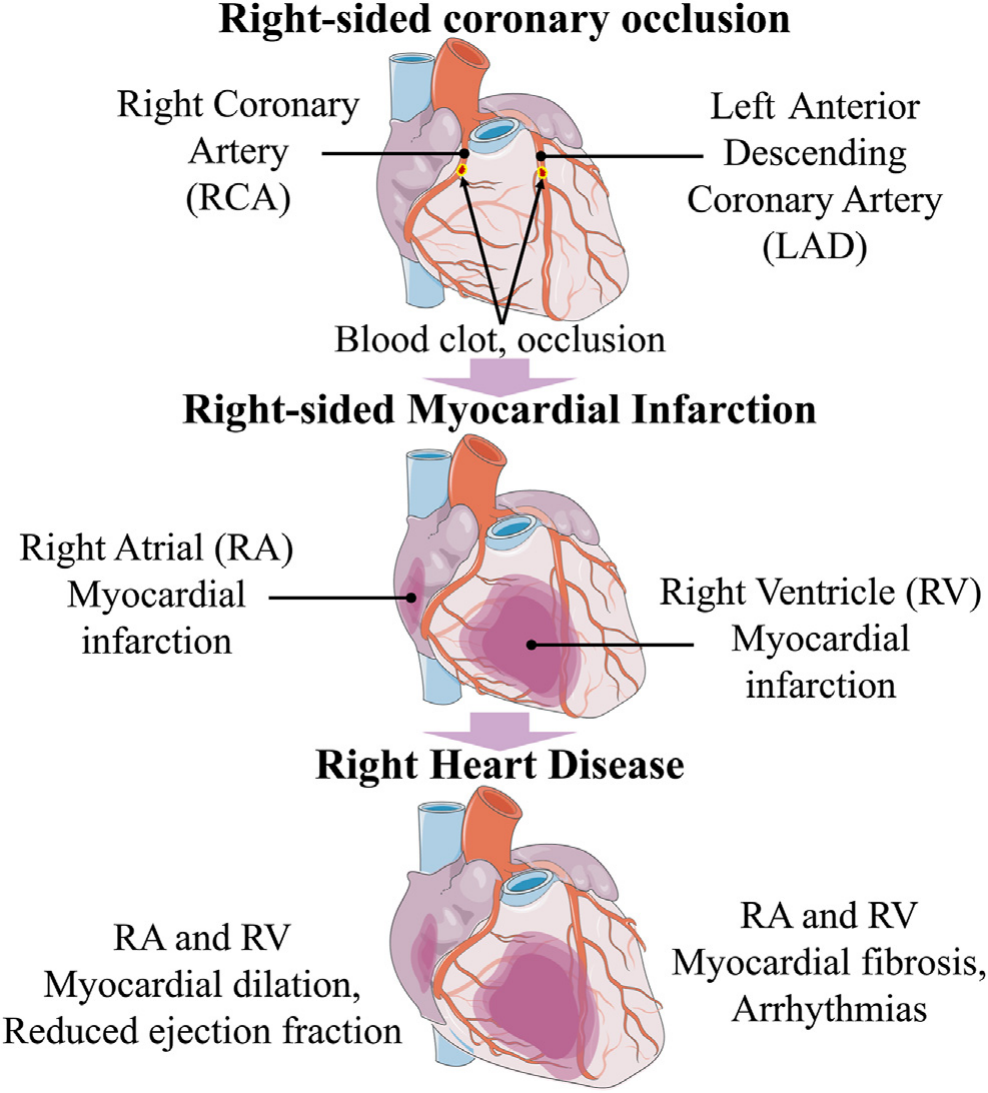
Right-sided coronary occlusion-induced myocardial infarction and right-sided heart disease. The right ventricle (RV) is irrigated by the right coronary artery and the left anterior descending coronary artery. If thrombosis or a blood clot obstructs the right coronary artery or/and LAD, the RV and right atrium (RA) will undergo myocardial ischemia and infarction. Myocardial infarction is accompanied by myocardial fibrosis, leading to RV and RA reduced function and dilation. Such RV and RA remodelling is an important substrate for cardiac arrhythmias in right-sided heart disease.

When a condition or blood clot provokes the obstruction of the right coronary artery, the left anterior descending artery, or/and the left circumflex artery (in patients with left circumflex artery dominance), perfusion and oxygenation of the right atrium and right ventricle will be interrupted, leading to myocardial hypoxia and ischemia.^49^, ^50^, ^51^, ^52^, ^53^, ^54^ Such right-sided cardiac hypoxia can provoke RV and RA myocardial infarction, activation of inflammation, the development of fibrosis, and reduced function^49^, ^50^, ^51^, ^52^, ^53^, ^54^, ^55^ (Fig. 4).

As the right ventricle supplies blood to the lungs, RA and RV ventricular electrical dysfunction can affect cardiac rhythm, and if the hemodynamic parameters are abnormal, the pulmonary circulation can be affected.^56^ Although cardiac hemodynamic function can be preserved in right-sided myocardial infarction,^57^ RA infarction, although it is less common, can impair right-sided cardiac electrical conduction.^58^, ^59^, ^60^ In severe RV dilation and infarction, the risk of reduced forward flow into the pulmonary artery trunk is elevated, and RHF can be accompanied by a drop in RV cardiac output.^61^, ^62^, ^63^ Infarction in the right side of the heart can lead to arrhythmias, mainly due to ischemia, fibrosis, and scar-tissue formation.^14^^,^^64^^,^^65^ The presence of fibrosis is recognized as an important contributor to perturbed electrical conduction, leading to an increased risk of conduction slowing, re-entrant circuit, and tachyarrhythmias, including flutters, AF, and ventricular fibrillation.^66^ Ventricular fibrillation and AF are associated with the risk of clot, due to abnormal ejection of blood, increasing the risk of aggravation of coronary occlusion, myocardial infarction, stroke, and sudden death.^64^^,^^67^ More investigation is required to identify the ratio of cardiac arrhythmias originating from right-sided myocardial infarction, to better describe the epidemiology and characterize the specific pathophysiology of AF and ventricular fibrillation in myocardial infarction-induced RHD.

**Figure 5:**
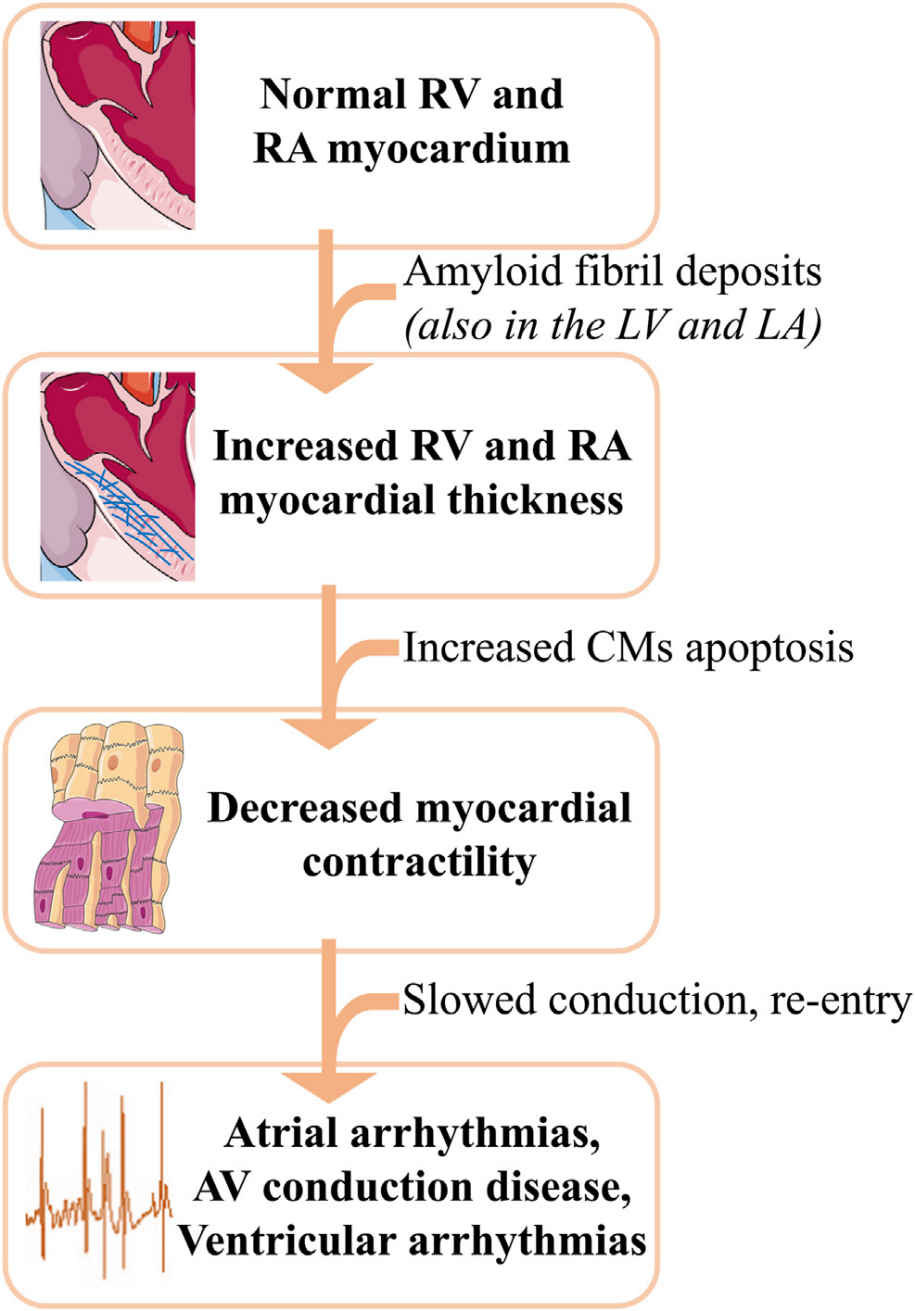
Amyloidosis in the pathogenesis of RHD and cardiac arrhythmias. Cardiac amyloidosis is characterized by the accumulation of abnormal amyloid fibril deposits in the left and right myocardium. Although this condition provokes LHD, it can affect the right ventricle (RV) and right atrium (RA), provoking increased thickness and stiffness of the myocardium. The presence of amyloid deposits perturbs cardiomyocytes’ (CM) contractility and slows electrical conduction. Impaired conduction is a risk factor for reentrant circuits, increasing the risk of cardiac arrhythmias, including atrial fibrillation, atrioventricular (AV) block, and ventricular fibrillation.

Other conditions, such as amyloidosis, can provoke RHD and RHF.^68^ Amyloidosis is a specific condition characterized by abnormal accumulation of amyloid proteins in the left and right myocardium, disrupting normal cardiac function.^68^^,^^69^ Cardiac amyloidosis, particularly transthyretin amyloid cardiomyopathy, is more prevalent in men than in women.^70^ However, women are often diagnosed at an older age and with more subtle cardiac symptoms.^71^ Although cardiac amyloidosis affects the left-sided function of the heart, it also should be suspected in any patient who presents with restrictive cardiomyopathy and prominent signs of RHF.^72^^,^^73^ Amyloidosis, including immunoglobulin light-chain amyloidosis, can provoke congestive heart disease, diastolic dysfunction, decreased tricuspid valve motion, right-heart chambers dilation, and hypertrophy associated with an increased risk of RHF^68^, ^69^, ^70^, ^71^, ^72^, ^73^, ^74^, ^75^ (Fig. 5).

In the context of myocardial infarction and amyloidosis, necrotic and dysfunctional areas lead to chaotic atrial conduction and increased vulnerability to RV and RA arrhythmias, including ventricular fibrillation and AF.^68^, ^69^, ^70^, ^71^, ^72^, ^73^, ^74^, ^75^

### RHD associated with congenital heart disease

In some adult patients with congenital heart disease, the right ventricle is subjected to failure due to increased pressure and volume overload.^76^ An estimated 10% of adult patients with congenital heart disease develop pulmonary arterial hypertension, with a mean pulmonary arterial pressure above 25 mm Hg measured during right-heart catheterization at rest.^41^ Atrial and ventricular septal defects and patent ductus arteriosus (ie, a persistent connection between the aorta and pulmonary artery) are common forms of congenital heart disease associated with pulmonary arterial hypertension and RV hypertrophy.

In congenital heart disease, left-to-right shunt creates chronic high pressure affecting the right ventricle and the pulmonary circulation, which can provoke pulmonary arterial hypertension.^42^^,^^43^ As described above, such chronic elevation of pressure provokes RV and RA structural and functional remodelling in congenital heart disease.^77^ Studies suggest that atrial arrhythmias are the most common complications in adults with congenital heart disease.^77^ The development of AF is responsible for a prevalence of 11% heart failure in young patients with congenital heart disease.^78^

### RHD associated with inherited genetic disorders and arrhythmogenic RV cardiomyopathy

Arrhythmogenic RV cardiomyopathy (ARVC) is an inherited disorder caused by genetic variants, affecting mainly the desmosomes.^79^ Although studies suggest that men and women have equal genetic inheritance of the disease, ARVC phenotypically seems to affect men more frequently and more severely than it does women.^80^ Studies suggest that a male predominance occurs in clinically diagnosed ARVC, with male-to-female ratios often ranging from 2:1 to 3:1.^81^ In ARVC, myocyte-to-myocyte adhesion can be impaired due to desmosomal mutations. Such abnormality progressively leads to arrhythmogenesis due to replacement of affected RV muscle with fibrous tissue. This structural abnormality can lead to ventricular arrhythmias and heart failure.^82^

The identified desmosomal gene variants responsible for ARVC include the following: plakoglobin, which affects about 3.8% of ARVC patients; plakiphilin-2 (*PKP2*), detected in 21%-48.9% of ARVC patients; desmoplakin, carried by 4.4%-14.3% of ARVC patients; desmoglein-2 (*DSG2*), present in 0.5%-3% of patients with ARVC; and desmocollin-2, observed in 3%-20% of ARVC patients.^83^^,^^84^

Nondesmosomal gene variants associated with ARVC include the following: transmembrane protein 43, carried by 0.4%-2.3% of ARVC patients; phospholamban (*PLN-p.Arg14del variant*), detected in 0.5%-5% of ARVC patients; desmin, with a prevalence of 0.9% in ARVC patients; filamin C, present in 1.8%-3% of ARVC patients; and cadherin-2, which affects < 1% of ARVC patients.^83^^,^^84^

ARVC is associated with severe perturbation of the electrical conduction of the right ventricle and is accompanied by the formation of fibrous areas in the RV myocardium.^85^ Hence, right-sided ventricular arrhythmia is commonly observed among ARVC patients.^86^ Progressive dysfunction of the right ventricle leads to RA electrical remodelling and dilation associated with increased risk of atrial arrhythmias, including AF.^87^ A study has shown that 14% of patients with ARVC have AF, and that 0.7% of genotype-positive patients develop AF.^87^ In a report involving patients with a *PKP2* deletion, the authors reported onset of AF.^88^ More investigation is required to better understand whether genetic variants observed in ARVC also are responsible for atrial remodelling and AF. Mainly, ARVC is accompanied by ventricular arrhythmia, and severe complications of ARVC include ventricular fibrillation and sudden death.^86^^,^^87^ To help clinicians assess the risk of ARVC-induced life-threatening events and guide treatment decisions, ARVC ventricular arrhythmia risk calculators have been developed.^89^ The most prominent and widely validated risk calculator is the one developed by Cadrin-Tourigny et al.^89^^,^^90^ This tool estimates the 1-year, 2-year, and 5-year risk of sustained ventricular arrhythmias in patients with a definite ARVC diagnosis. The main factors included in this calculator are as follows: patient demographics (age at diagnosis, sex, clinical history, prior cardiac syncope, history of nonsustained ventricular tachycardia, electrocardiogram detection of premature ventricular complexes, number of inverted T-waves, and echocardiography assessment of RV ejection fraction.^89^^,^^90^

Brugada syndrome (BrS) is another type of inherited RHD.^91^ BrS is primarily an electrical heart disease (channelopathy)^92^ BrS is characterized by abnormalities in the cardiac electrical activity, specifically related to ion channels and most commonly the sodium channel via the gene coding the sodium channel protein type 5 subunit alpha (*SCN5A* gene).^91^ BrS is identified by a characteristic electrocardiogram pattern (ST-segment elevation in the right precordial leads) and an increased risk of ventricular arrhythmias and sudden cardiac death, often in people with seemingly structurally normal hearts.^93^

## Mechanisms of Cardiac Arrhythmia in RHD

The collective knowledge about cardiac arrhythmia mechanisms is constantly increasing, thanks to advances in clinical and experimental research aiming to understand the mechanisms underlying the association between RHD and cardiac arrhythmias.

Studies have shown that the prevalence of AF is 7% in tetralogy of Fallot^94^ and approximately 20% in pulmonary hypertension.^12^^,^^13^ A report suggested that 75% of patients with adult congenital heart disease develop atrial flutters.^95^ Studies have suggested that the predominant pathogenetic abnormality in BrS affecting the *SCN5A* gene is associated with predisposition to ventricular tachycardia and ventricular fibrillation.^96^^,^^97^ Mounting evidence suggests that electrical remodelling in BrS is accompanied with RV functional remodelling, including reduced RV outflow track conduction.^98^^,^^99^

In this context, the understanding of the cellular and structural mechanisms underlying the development of cardiac arrhythmias in RHD is crucial to development of new strategies of treatment to ameliorate patient management and quality of life.

### Right-sided cardiac hypertrophy, dilation, and conduction abnormalities

In RHD, the right ventricle is subjected to structural remodelling, including progressive myocardial hypertrophy.^100^ RV hypertrophy is compensatory in the early and intermediate stages of RV pressure and/or volume overload.^40^ Over time, this compensatory mechanism becomes progressively insufficient, leading to RV dilation and failure.^101^ Hypertrophied and stretched cardiomyocytes become mechanically and electrically inefficient.^102^ Dysfunctional RV cardiomyocytes then may undergo cell death due to apoptosis, necrosis, and other cell-death pathways, leading to tissue fibrosis and altered gap-junction structure and function.^66^^,^^103^^,^^104^ The loss of viable cardiomyocytes and replacement by fibrotic tissue disrupts RV conduction and reduces subendocardial electrogram amplitude, manifesting as low-voltage zones on electroanatomic mapping.^104^, ^105^, ^106^ Dysfunctional and dying cardiomyocytes activate damage-associated molecular patterns, oxidative stress, and inflammation.^107^ If unresolved, chronic myocardial inflammation promotes the development of fibrosis, which aggravates conduction abnormalities, increasing the risk of reentry and cardiac arrhythmias, including ventricular tachyarrhythmia and/or ventricular fibrillation due to anatomic reentry circuits anchored by myocardial scars.^14^^,^^107^ The RV dilation occurring with advanced RHD is accompanied by deformation of the tricuspid valve annulus and tricuspid regurgitation that further worsens RV dysfunction.^108^^,^^109^ Tricuspid valve annular deformation is accompanied by a decrease in the tricuspid annular plane systolic excursion, an important parameter in the assessment of RHD.^108^^,^^109^ RV dilation and functional tricuspid regurgitation induce chronic RA volume overload, provoking a cascade of deleterious events in the right atrium, marked by myocardial hypertrophy, dilation, inflammation, and fibrosis, leading to refractoriness, reentry, and an increased risk of cardiac arrhythmia, including atrial flutter and AF.^13^^,^^108^, ^109^, ^110^, ^111^ In patients with RHD, myocardial fibrosis can occur, due to either the above-described remodelling, or the development of scars as a result of repair surgeries in patients with congenital heart disease or tetralogy of Fallot.^94^ Patients who have received atriotomies often develop intra-atrial reentrant tachycardias, including atrial flutter.^77^^,^^94^ Congenital heart disease patients treated by RV surgical incisions or patches often develop macro-reentrant ventricular arrhythmias, including ventricular fibrillation.^77^^,^^94^^,^^112^

### RHD-associated cellular remodelling in cardiomyocytes, fibroblasts, and endothelial cell dysfunctions and inflammation

A rat model of RHD induced by pulmonary artery trunk banding has shown that although fibroblasts are involved in the inflammatory response to cardiac insult in RHD, cardiomyocytes also contribute to this inflammatory status via the activation of biomarkers such as the NLRP3 (nucleotide-binding domain, leucine-rich-containing family, pyrin domain-containing-3 inflammasome), interleukin (IL)-6 and IL-1β.^113^

**Figure 6:**
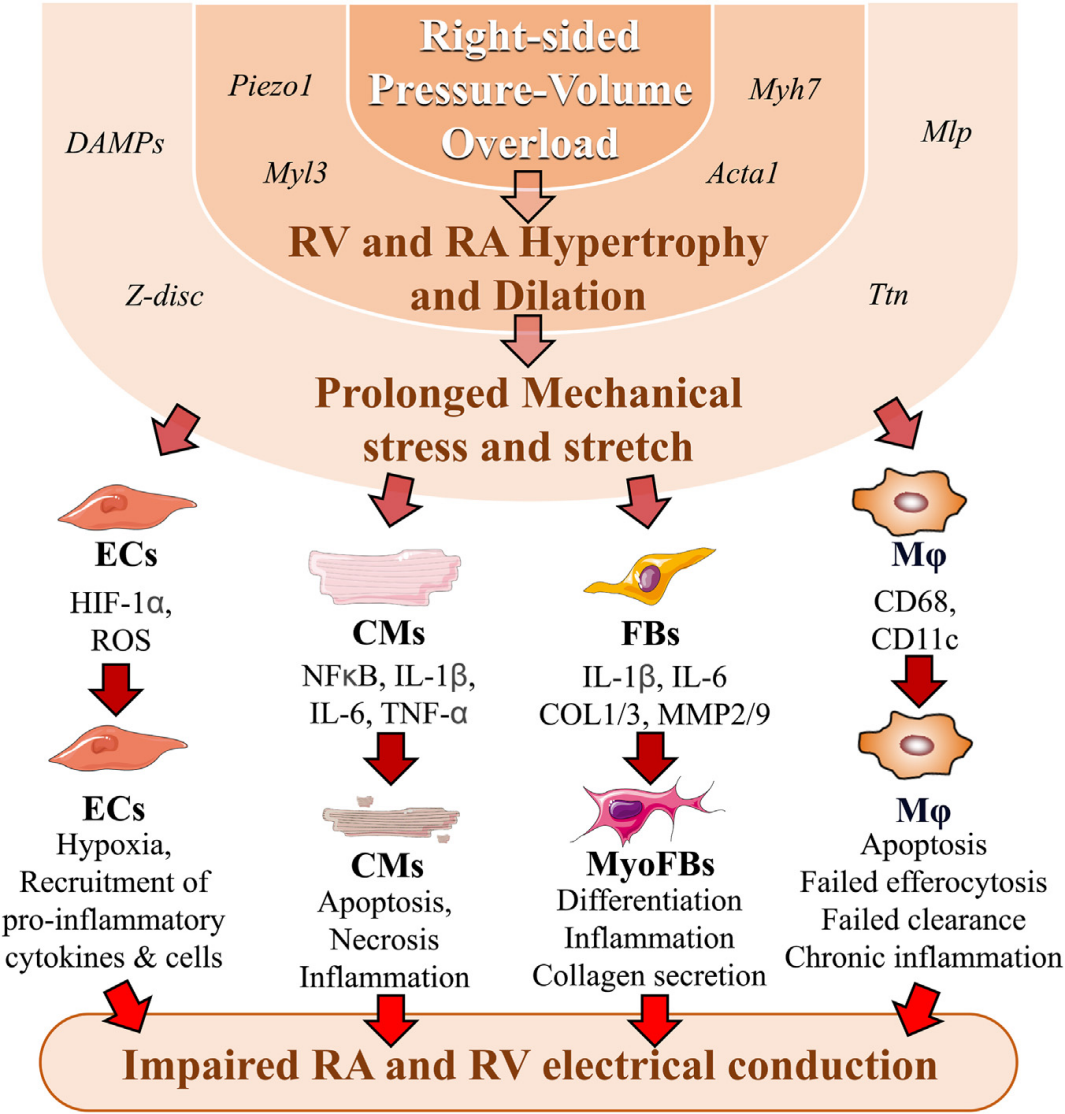
Right-heart disease –induced arrhythmogenic cellular remodelling. Conditions generating right-sided pressure-volume overload can provoke right ventricular (RV) and right atrial (RA) dilation and hypertrophy. Genes involved in such remodelling include mechanosensitive ion channel component 1 (*Piezo1*)*,* myosin light chain 3 (*Myl3*)*,* myosin heavy chain 7 (*Myh7*)*,* and actin alpha 1 (*Acta1*). Progressive RV and RA dilation and hypertension exert prolonged mechanical stress and stretch on the cells structuring the right ventricle and right atrium. Markers of mechanical stretch and stress include muscle lim protein *(Mlp),* titin *(Ttn),* z-lines z-bands *(Z-disc),* and damage-associated molecular patterns (DAMPs) signals. The endothelial cells (ECs), cardiomyocytes (CMs), fibroblasts (FBs), and macrophages (M**ϕ**) respond to mechanical stretch and stress by activating specific cells involved in hypoxia (ECs: hypoxia-inducible factor alpha [HIF1α], reactive oxidative species [ROS]); inflammation (CMs: nuclear factor kappa B [NFkB], interleukin (IL)-1β, IL-6, tumor necrosis factor alpha (TNFα); M**ϕ**: cluster of differentiation 68 [CD68], CD11c); and fibrosis (FBs: collagen 1 and 3 [COL1/3], matrix metalloprotein 2 and 9 [MMP2/9]). Progression and maintenance of the disease can lead to the following: (i) EC-induced recruitment of proinflammatory cytokines and cells; (ii) CMs apoptosis and necrosis promoting RV and RA inflammation; (iii) FBs differentiation into myoFBs, leading to further inflammation and development of fibrosis; (iv) proinflammatory M**ϕ** recruitment and apoptosis, which is accompanied with failed mechanisms of efferocytosis and clearance, contributing to maintenance of the inflammatory status. Altogether, the cellular response to right-sided pressure and/or volume overload interferes with normal electrical conduction, provoking RV and RA conduction slowing and vulnerability to cardiac arrhythmias. MyoFBs, myo-fibroblasts.

RA dilation and mechanical stretch can provoke the activation of inflammation-signaling cascades, such as nuclear factor kappa B,^114^^,^^115^ which is a transcription factor that regulates the expression of proinflammatory cytokines such as IL-6, tumor necrosis factor-alpha (TNF-α), IL-1β, and IL-18^115^ (Fig. 6).

These inflammatory stimuli excreted by the cardiomyocytes have paracrine effects via their activation of neighbor cell types, including the fibroblasts.^116^ In RHD, activated fibroblasts from the right ventricle and right atrium will differentiate into myofibroblasts and also start to secrete proinflammatory cytokines.^117^^,^^118^ In addition, myofibroblasts excessively secrete collagen biomarkers (collagen-1, collagen-3) and matrix metalloproteinases-2 and -9, contributing to buildup of the extracellular matrix, which constitutes myocardial fibrous areas responsible for low-voltage zones in the right ventricle and right atrium^116^, ^117^, ^118^, ^119^ (Fig. 6).

In response to RHD-induced remodelling, endothelial cells also are affected.^120^ A well accepted finding is that endothelial cells contribute to the activation and aggravation of myocardial hypoxia in response to cardiac disorders.^120^, ^121^, ^122^, ^123^ In RHD, endothelial cells also may participate in the recruitment of immune cells from the bloodstream by facilitating their migration to the right ventricle and right atrium.^120^, ^121^, ^122^, ^123^, ^124^ Recruited cells include proinflammatory macrophages, which have been demonstrated to contribute to the myocardial inflammatory status in RHD.^125^^,^^126^

Inflammation is a normal biological process that is activated to signal damage or injury.^118^ Inflammation aims to promote repair of damaged tissues and must be followed by inflammation termination and homeostasis.^118^^,^^127^^,^^128^ In progressive conditions such as RHD, the right ventricle and the right atrium have been shown to display a chronic and unresolved inflammatory status, which leads to cardiac fibrosis and arrhythmogenesis.^13^^,^^14^^,^^107^^,^^118^^,^^127^, ^128^, ^129^, ^130^ Nevertheless, an important point to note is that although atrial fibrosis is a major contributor to cardiac arrhythmias, including AF, some patients with cardiac fibrosis might not experience rhythm disorders.^131^ As described above, the presence of fibrosis indicates an underlying structural remodelling process driven by various factors, including cellular inflammation, hypoxia, and apoptosis.^132^ Cardiac arrhythmias are influenced by complex convergence of other triggers, including ectopic beats, chamber dilation, ion-channel remodelling, and autonomic nervous system activity.^133^ Hence, a patient can have a fibrotic substrate due to chronic RHD-associated hypertension and ventricular dysfunction, although the specific electrical triggers and remodelling required for the initiation and maintenance of arrhythmias including AF might not be present or sufficient.^134^ Another possibility is that the fibrosis might appear primarily in the right and left ventricle, contributing to heart failure, without the atrial changes necessary for atrial arrhythmogenesis, suggesting a different vulnerability to ventricular vs atrial arrhythmias.^134^

### Electrical remodelling provoked by RHD-associated RV and RA inflammation and fibrosis

The cellular mechanisms of RHD-induced inflammation involve a complex interplay among mechanical stress, neurohormonal activation, immune cell recruitment, and molecular signaling pathways.^135^ In RHD, RV and RA hypertrophy and dilation generate mechanical stretch that activates proinflammatory pathways in the cardiomyocytes, endothelial cells, and fibroblasts.^113^^,^^118^^,^^122^ Stretch-sensitive receptors and integrins expressed in RV and RA cardiomyocytes detect prolonged mechanical stretch, leading to dysregulation of the calcium (Ca^2+^)-handling machinery, perturbing cardiomyocytes’ contractility.^136^

RV and RA cell remodelling induced by the progression of RHD leads to chronic and unresolved inflammation and fibrosis responsible for cardiac arrhythmias.^113^^,^^118^^,^^126^^,^^127^ Due to the multiple cell types impacted by the progressive remodelling in RHD, inflammatory signals are exacerbated and unresolved, promoting chronicity of the inflammatory status.^126^^,^^127^ Such an inflammatory profile might stimulate the aggravation of the surface occupied by fibrotic tissue in the right ventricle and right atrium in RHD.^118^ Ventricular and atrial fibrous content disrupt the normal architecture of the myocardium, leading to impaired electrical conduction due to decreased activity and lateralization of gap junctions.^137^^,^^138^ A rat model of RHD induced by pulmonary artery trunk banding has shown that myocardial fibrosis was accompanied with electrical remodelling affecting the atrial cardiomyocytes via diminution of expression and increased lateralization of connexin 43.^113^

Studies have shown that in RHD, the right ventricle and right atrium are affected by decreased expression and activity of voltage-gated potassium channels and Ca^2+^-activated potassium channels involved in the repolarization phase of the action potential, which contributes to early-after depolarization and arrhythmia.^139^, ^140^, ^141^, ^142^ In addition, the inward sodium (Na^+^) current was shown to be increased in RHD via increased voltage-gated Na^+^ channels promoting depolarization, automaticity, and ectopy.^113^^,^^143^^,^^144^

The compensative phase of RHD characterized by RV and RA hypertrophy is often accompanied by increased activity of the inward Ca^2+^ channels, contributing to increased Ca^2+^ intracellular concentration, cardiomyocyte hyperreactivity, and cardiomyocyte hyper-contractility, which promote arrhythmogenesis.^145^ Experimental studies have shown that decreased expression of sarcoplasmic and/or endoplasmic reticulum Ca^2+^-adenosine triphosphatase^146^^,^^147^ and mutations affecting *Scn5a* expression in animal models of RHD can lead to RA remodelling and AF.^148^ This important electrical remodelling might help explain the typical prolonged action-potential duration observed in RHD patients.^66^^,^^113^^,^^142^ The gene variants associated with gain-of-function of the *Scn5a* gene, which encodes the Nav1.5 sodium channel, can lead to a prolongation of the plateau, in addition to a slower and prolonged repolarization phase, due to an increase in late Na^+^ current (as seen in long QT syndrome type 3), and therefore, provoke a longer action-potential duration.^148^ Such electrical changes contribute to the occurrence of early afterdepolarizations.^14^^,^^149^

**Figure 7:**
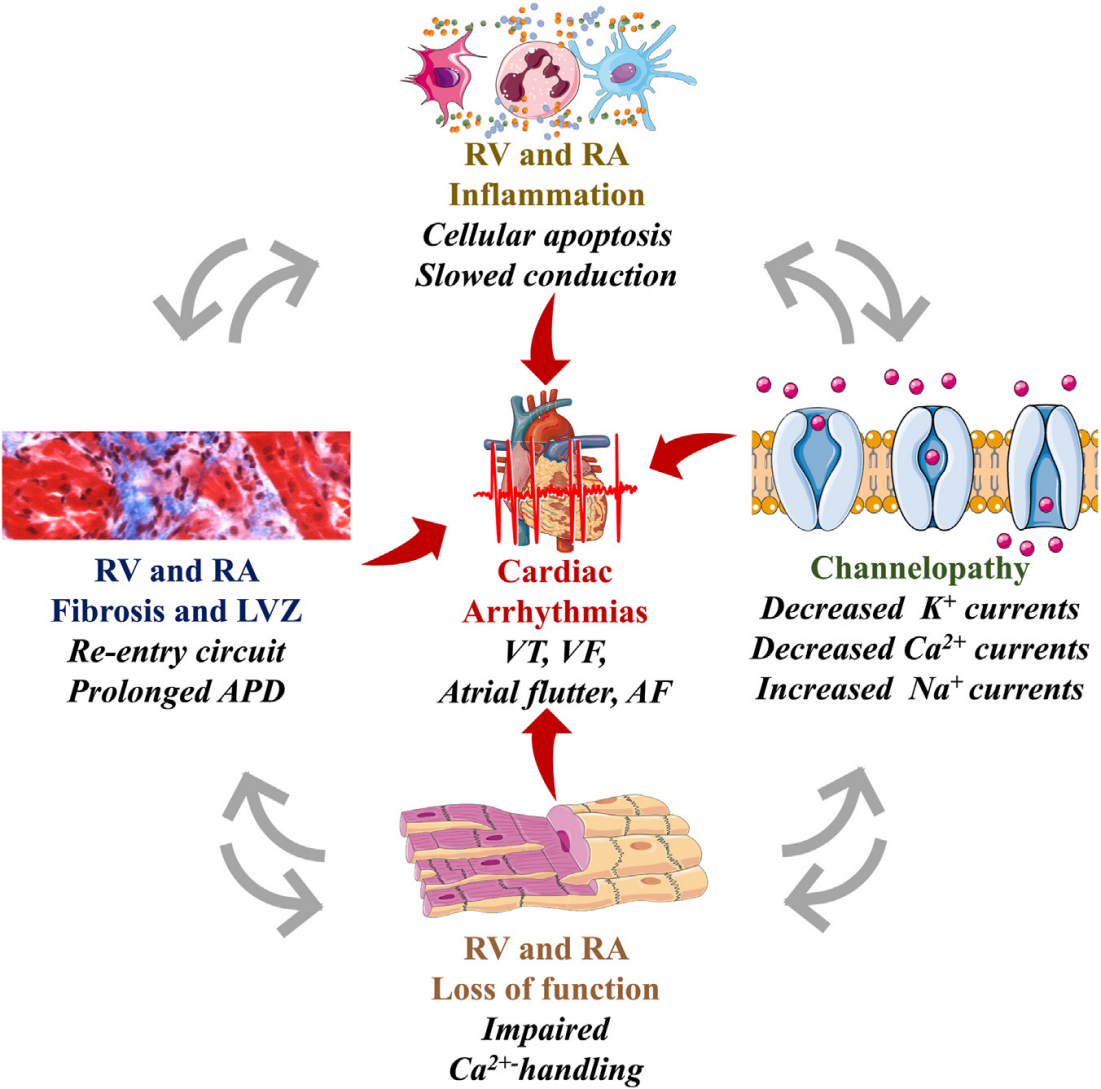
Right-heart disease (RHD)-induced tissular inflammation and fibrosis in right ventricular (RV) and right atrial (RA) arrhythmogenesis. Structural and functional remodelling affecting the right ventricle and right atrium in RHD lead to chronic inflammation, fibrosis, low voltage zones (LVZ), impaired RV and RA functions, and abnormal electrical conduction via reentry circuit, decreased potassium (K^+^) and calcium (Ca^2+^) channel activity, and increased inward sodium (Na^+^) currents. These RHD-induced tissular and electrical changes contribute to prolonged action potential duration (APD), and constitute a substrate for cardiac rhythm disorders occurring in the right ventricle (ventricular tachyarrhythmia [VT] and fibrillation [VF]) and the right atrium (atrial flutter and atrial fibrillation [AF]).

In RHD, during the decompensated phase, in which the right ventricle and right atrium are irreversibly dilated and hypertrophied, the above-described electrical remodelling coupled with inefficient Ca^2+^-orchestrated contractile machinery increases the risk of RHF, AF, ventricular fibrillation, and sudden death^113^^,^^144^^,^^146^ (Fig. 7).

## New Investigation Insights: Experimental Models of RHD, Potentially Relevant for Cardiac Arrhythmia Studies

Experimental models are important to better elucidate the mechanisms underlying the development of cardiac remodelling and arrhythmias in RHD. Researchers have employed various *in vitro* and *in vivo* models, to mimic specific aspects of RHD, clarify the pathophysiological mechanisms, and propose innovative therapeutic strategies.^150^^,^^151^

### Cellular models

Cellular models, especially induced pluripotent stem-cells (iPSC) reprogrammed from patients to become cardiomyocytes, provide tools for patient-specific disease modelling and drug screening at the cellular level, and animal models allow for the study of disease progression and therapeutic efficacy in a whole-organ context.^152^ Cellular models have contributed to a better understanding of the molecular and electrophysiological aspects of the pathophysiology of RHD.^152^

Heterologous expression systems have been explored in noncardiac cell lines, such as HEK293 cells, CHO cells, and Xenopus oocytes, and found to express specific mutant ion channels (eg, *SCN5A* for BrS) or desmosomal proteins (eg, *PKP2* for ARVC).^153^^,^^154^

Patient-specific iPSC-cardiomyocytes reprogrammed from somatic cells (eg, skin fibroblasts, blood cells) collected from ARVC or BrS patients carry the donor's specific genetic mutations and can recapitulate disease-specific phenotypes *in vitro*. In cellular models of iPSC-cardiomyocytes, the following have been possible: description of the reduced desmosomal protein expression and abnormal localization of targeted proteins involved in ARVC^155^; study of the disrupted cell-cell junctions and gap junction abnormalities (eg, reduced connexin43) typical in RHD^156^; recapitulation of characteristic electrophysiological abnormalities such as the reduced sodium current (INa^+^) observed in BrS^157^; characterization of impaired cardiomyocytes contractile properties and altered Ca^2+^-handling^158^; evaluation of the efficacy of new medications, such as sodium-channel blockers and antiarrhythmic drugs such as quinidine in BrS^159^; and/or testing of gene-replacement therapies, as, for example, to promote *PKP2* delivery to restore *PKP2* expression and function in ARVC.^160^

The main limitations of iPSC-cardiomyocytes reside in the fact that they are immature phenotypes compared to adult cardiomyocytes, and in the 2-dimensional culture environment, which may not fully replicate the 3-dimensional cardiac tissue architecture.^161^ Efforts to create 3-dimensional engineered heart tissues from iPSC-cardiomyocytes are currently in development to address this issue.^162^

### Monocrotaline

The monocrotaline model has been used widely to provoke pulmonary arterial hypertension-induced RHD.^163^^,^^164^ With a single dose of 60 mg/kg monocrotaline injected intraperitoneally, severe pulmonary arterial hypertension can be induced in rats in 3-4 weeks postinjection.^163^^,^^164^ Studies have shown that monocrotaline-induced RHD is associated with increased vulnerability to ventricular and atrial arrhythmias, compared to control.^14^^,^^165^ An investigation of the impact of inflammation-resolution in the prevention of AF has shown that daily treatment with resolvin-D1 can attenuate RA fibrosis in monocrotaline-induced RHD.^165^ In addition, a study has demonstrated that ventricular fibrosis and ventricular arrhythmias can be prevented by the administration of dapagliflozin, a sodium-glucose cotransporter 2 inhibitor, which was shown to attenuate the expression and activity of nuclear factor-kappa B.^166^ When studying the role of inflammation in RHD-induced arrhythmogenesis, an important limitation of the monocrotaline model is the development of pneumo-toxicity, which might influence the cardiac inflammatory status observed in this model^14^^,^^165^ (Table 1).

**Table 1:** Available experimental models for the comprehension of cardiac arrhythmias in right-heart disease (RHD)

Experimental models of RHD	Experimental requirements for reproducible RHD induction	Potential arrhythmogenic RV and RA remodelling promoted	Limitations	Studied cardiac arrhythmias or arrhythmogenic substrate	Refs.
Cellular models	*In vitro* culture of cells (eg, HEK293; iPSC-CMs)	Used to express specific gene variants involved in RHD-specific genotype and phenotype (eg, *SCN5A, PKP2*)	Limited to cellular aspects and 2D environment; *in vitro* methods do not help to reproduce the systemic impact of the specific RHD-associated gene variants	↓ CMs-CMs gap-junctions ↑ CMs contractility ↓ Eletrical conduction	152, 153, 154, 155, 156, 157, 158, 159
MCT	Single i.p. injection of 60 mg/kg; observe cardiac remodelling in 3-4 wk post-injection	PAH-induced RV overload; RV and RA hypertrophy and dilation; RV and RA inflammation might be exacerbated by MCT-induced lung disease	Requires careful interpretation of RV and RA inflammatory profile, because circulating inflammatory signals originating from MCT-induced pneumotoxicity may influence the right myocardial inflammation	↑ AF ↑ Atrial flutter ↑ VF	14, 163, 164, 165, 166
PAB	Perform a permanent reduction of the PAT diameter of 60% or above, to observe RHD associated with arrhythmogenic changes in 3-4 wk; A precise selection of the ligation’s diameter according to the animal weight and echography monitoring for validation is required	PAB-induced RV overload; RV and RA hypertrophy and dilation; The RV and RA inflammation and fibrosis are due to only the PAB-induced RHD, as the lungs are not affected	The method is appropriate to study specific impact of PAT obstruction on RHD; this approach might not be relevant to understand the pathophysiological mechanisms related to the progression of diseases, because pulmonary function is not affected in PAB	↑ AF ↑ Atrial flutter ↑ VF	14, 113, 167
SuHx	Single injection of Sugen-5416 (20 mg/kg) and housing in a hypoxic chamber for 3-4 wk	SuHx-induced RV overload; RV and RA hypertrophy, dilation, fibrosis and inflammation	The SuHx model requires careful interpretation of the RV and RA inflammatory profile, due to the underlying lung disease	↑ VF	126, 171, 172, 173, 174
RCA ligation	The micro-surgical intervention must be performed carefully to ligate the RCA, while avoiding rupture and hemorrhage; Vulnerability to cardiac arrhythmia can be tested 3-4 wk post-suture; RV infarction obtained with suture of the RV branch of the LAD, but the induced septal and apex infarction might not be relevant to the typical RHD	RV and RA ischemia associated with myocardial hypoxia, inflammation, and fibrosis	Might not be relevant to study the hypertrophic phase in RHD; interesting to study right-sided fibrosis, MI, and RHF	↑ AF	168, 169
Transgenic mice	*Fog2*^*+/+*^ mice	Induction of HF; ventricular dysfunction; notRV-specific, but constitutes a relevant biventricular model of HF	Overexpression of a non-cardiac-specific gene might provoke systemic remodelling; Such nonspecificity can affect other organs that are involved in arrhythmias; this suggests that careful interpretation is required of the association between induced cardiac disease and observed arrhythmias	↑ AF	176
	*Tnfa*^*+/+*^ mice	Reduction of EF Ventricular fibrosis CM apoptosis		↑ AF	177, 178, 179
	*Pkp2*^*-/-*^ mice	Reproduce some aspects of the ARVC phenotype Provokes desmosomal dysfunction Leads to abnormal Ca^2+^-handling		↑ VAs	180, 181, 182
	*DSG2*^*+/+*^ mice				
	*SCN5A-/-* mice	Reproduce electrical phenotype observed in BrS; loss or Gain-of-function on sodium current		↑ VAs	183

AF, atrial fibrillation; ARVC, arrhythmogenic right-ventricular cardiomyopathy; BrS, Brugada syndrome; CM, cardiomyocyte; DSG2, desmoglein-2; EF, ejection fraction; Fog2, friend-of-GATA2 protein; HF, heart falure; i.p., intraperitoneal; iPSC-CM, induced pluripotent stem cell-derived cardiomyocytes; LAD, left anterior descending coronary artery; MCT, monocrotaline; MI, myocardial infarction; PAB, pulmonary artery banding; PAH, pulmonary arterial hypertension; PAT, pulmonary artery trunk; PKP2, plakophilin-2; RA, right atrium; RCA, right coronary artery; Refs., references, RHD, right-heart disease; RHF, right-heart failure; RV, right ventricle; SCN5a, sodium channel protein type 5 subunit alpha; SuHx, sugen hypoxia; Tnfα, transforming growth factor alpha; VA, ventricular arrhythmia; VF, ventricular fibrillation; 2D, 2-dimensional.

### Pulmonary artery trunk banding

Pulmonary artery banding consists of the permanent reduction of the pulmonary artery trunk diameter to provoke chronic RV pressure overload and RHD.^113^^,^^167^ In constrast to the above-described monocrotaline model, the pulmonary artery banding procedure does not provoke pulmonary inflammation.^113^^,^^167^ Hence, this approach is optimal for studying RV and RA inflammation resulting only from pulmonary artery banding–induced RHD.^113^^,^^166^ A recent study has shown that pulmonary artery banding–induced RHD is associated with RV and RA inflammation identified by increased expression of IL-1β and IL-6.^113^ In addition, pulmonary artery banding–induced RHD was associated with decreased expression of transverse tubule (T-tubule) proteins, such as bridging integrator-1, caveolin-3, and junctophilin-2, reported to promote arrhythmogenesis.^113^ Moreover, the impact of pulmonary artery banding–induced RHD on cardiomyocytes’ remodelling was shown to involve perturbation of the cardiomyocytes’ contractile machinery, slow atrial conduction, increase action potential duration, and increase AF vulnerability^113^ (Table 1).

### Right coronary artery ligation

The rodent model of right coronary artery ligation is an efficient approach to provoke RA and RV myocardial infarction and RHD. In a mouse model, right coronary artery ligation was associated with severe RV heart failure and interventricular remodelling.^168^ Although it is under-used in the study of RHD-induced arrhythmogenesis, right coronary artery ligation is an interesting tool for evaluating the impact of right-sided inflammation and fibrosis in the development of RHF and arrhythmias (Table 1). In a dog model, right coronary artery occlusion was associated with increased risk of AF.^169^ In contrast, left-sided myocardial infarction provoked by the ligation of the left anterior descending coronary artery is well described and reproducible, as used in developing the understanding of LV ischemia-induced arrhythmias.^170^ Recent results demonstrate that the strategy of inflammation resolution involving resolvin-D1 treatment can prevent AF induced by the ligation of the left anterior descending coronary artery.^127^ The specific impact and pathophysiology of right coronary artery ligation and right-sided myocardial infarction in AF remain poorly explored.

### Sugen hypoxia

To induce pulmonary hypertension in mice and rats, the antagonization of the vascular endothelial growth factor receptor 2 using Sugen, known as SU5416, coupled with housing in a hypoxic atmosphere (10% oxygen for multiple weeks), has been reported in numerous studies to be an efficient strategy to provoke lung hypoxia, pulmonary arterial hypertension, and RHD.^171^^,^^172^ The sugen hypoxia-induced right-sided remodelling includes decompensated RV failure characterized by RV and RA dilation, and decreased RV ejection fraction.^171^^,^^172^ The sugen hypoxia model has been shown to be associated with increased RV expression inflammatory (NLRP3, IL-1β) and fibrosis markers (collagen-1/3, matrix metalloprotein 2 and 9).^126^^,^^173^ Sugen hypoxia-induced RV fibrosis was accompanied by increased VF vulnerability, compared to control animals^174^ (Table 1).

### Transgenic mice

Transgenic mice have been used to generate heart failure, with the purpose of better understanding heart failure-induced cardiac arrhythmias and AF.^175^ Studies involving specific mutation of genes and proteins specifically targeting RV and RA remodelling are lacking. However, some investigations reporting biventricular changes have helped to clarify some aspects of the arrhythmogenic pathophysiology. Clinical and experimental studies have shown that the transcription factor FOG2 (friend of GATA2; encoded by *ZFPM2*) is upregulated in human and mice ventricles during heart failure.^176^ In transgenic mice overexpressing FOG2, heart failure was induced, and was characterized by atrial enlargement, ventricular dysfunction, and increased AF.^177^ In mice models of tumor necrosis factor-α (TNF-α) overexpression, severe reduction of ventricular ejection fraction was induced, accompanied by ventricular fibrosis, cardiomyocyte apoptosis, and AF.^178^^,^^179^ In ARVC mouse models mimicking the desmosomal gene variants observed in humans, *PKP2* knockout and *DSG2* knock-in have helped illuminate the following: the mechanisms of ARVC-specific RV (and sometimes LV) dilation and dysfunction; myocardial fibrosis; ventricular arrhythmias and sudden cardiac death; disruption of desmosomes and gap junctions; and abnormal Ca^2+^-handling.^180^, ^181^, ^182^ Transgenic mice with *SCN5A* mutations have made possible a better understanding of the pathophysiology of BrS by reproducing the electrophysiological phenotype involving reduction of sodium currents and enhanced susceptibility to ventricular arrhythmias.^183^

Although most transgenic models of heart failure are not specific and provoke either LV or biventricular remodelling, they constitute interesting tools in the understanding of heart failure-induced arrhythmogenesis (Table 1). More research is required to develop specific models of right-sided cardiac remodelling to contribute to the improvement of scientific knowledge about the association between RHD and cardiac arrhythmias.

## Future Directions

### Inflammation resolution

Recent studies have shown that patients with RHD and cardiac arrhythmia carry elevated plasmatic levels of inflammatory markers, including IL-6.^184^^,^^185^ In monocrotaline and pulmonary artery banding models of RHD and arrhythmias, elevated levels of inflammatory cytokines were reported in the right atrium and right ventricle.^14^^,^^113^^,^^127^^,^^185^ These data suggest that inflammation is a potential target in the prevention and treatment of cardiac rhythm disorders in RHD.^113^ An interesting finding to note is that daily treatment with resolvin-D1 was shown to prevent RA inflammation and AF susceptibility in monocrotaline-induced RHD.^113^ Further investigations are required to better characterize the impact of strategies promoting inflammation resolution in RHD and cardiac arrhythmias (Table 2).

**Table 2:** Potential future therapeutic strategies in management of cardiac dysrhythmias in right heart disease (RHD)

New treatments in RHD and AF management	Potential role on RHD	Potential role on cardiac arrhythmia	Refs.
Inflammation-resolution	Prevent RHD-induced inflammation and fibrosis, but not RV and RA hypertrophy (ie, *RvD1*)	Decrease AF vulnerability	127, 165
Gene-therapy	Prevent MCT-induced RV malfunction (ie, *AAV9-JPH2*) Prevent the expression of gene-variants affecting desmosomal (ie, *AAV9-PKP2*) and non-desmosomal proteins (ie, *AAV9-PLN-R14del*) involved in ARVC	Rescue cardiac contractility, reduces Ca^2+^ spark. Prevent malfunctional PLN and SERCA2a activity. Normalizes Ca^2+^-handling	188 180, 181
Personalized medicine	Identification of specific genes involved in RHD-induced RV and RA remodelling Identification of specific genes involved in ARVC and promotion of their expression via gene therapy Clinical trials ongoing, including NCT06228924; NCT06109181	Potential for precise selection of drugs in the management of AF in RHD patients Promote functional PKP2 gene and protein, normalized desmosomal function and attenuate the risk of ARVC	192, 193 195, 196, 197
Artificial intelligence	Prediction of RHD development Optimization of the choice of treatment strategies	Precise selection of prophylactic and curative treatments	194, 195, 196, 197, 198, 199, 200, 201, 202, 203, 204

AAV9, adeno-associated virus serotype 9; AF, atrial fibrillation; ARVC, arrhythmogenic right ventricular cardiomyopathy; Ca^2+^, calcium; JPH2, junctophilin 2; MCT, monocrotaline; PLN, phospholamaban; RA, right atrium; Refs., references; RHD, right heart disease; RV, right ventricle; RvD1, resolvin D1; SERCA2a, sarcoplasmic/endoplasmic reticulum Ca^2+^-adenosine triphosphatase.

### Gene therapy

The management of cardiac arrhythmia in the context of RHD is a complex therapeutic approach that requires the consideration of various comorbidities in selecting the appropriate medication and strategy, to avoid toxic combinations of medications. Recent studies have proposed that management of RHD be optimized by targeting the dysregulated genes specifically, using gene therapy to stimulate or optimize level of expression.^186^^,^^187^ In a mouse model of HF provoked by transverse aortic constriction-induced pressure overload, gene therapy involving adeno-associated virus type 9 (AAV9)-mediated overexpression of junctophilin 2 contributed to rescuing cardiac contractility by reducing Ca^2+^-spark frequency, and normalizing the sarcoplasmic reticulum Ca^2+^-handling machinery.^188^ In RHD, the knockdown of junctophilin 2 was associated with impaired mitochondrial function in RV cardiomyocytes.^189^ AAV9-mediated overexpression of junctophilin 2 rescued T-tubules mitochondrial morphology and improved RV function in a rat model of monocrotaline-induced RHD.^190^ In preclinical models of gene therapy involving AAV9 and CRISPR/cas9 strategies, disruption of the *PLN-R14del* mutation (which leads to abnormal PLN protein) has shown potential to prevent impairment of the sarcoplasmic and/or endoplasmic reticulum Ca2+-adenosine triphosphatase 2a activity, reduce disruption of Ca^2+^-handling, and attenuate ARVC pathogenesis^180^^,^^181^ (Table 2).

### Personalized medicine strategies

Individual genetic profile is well accepted to be an important tool to guide treatment decisions in the prevention and diagnosis of cardiac disorders.^191^ Precision medicine has emerged as a powerful approach to specifically target the molecules involved in the development of diseases in a personalized manner.^192^ In RHD, transcriptomic analysis has helped to elucidate the specific genes involved in RHD.^193^ Experimental studies have revealed genes that are remodelled in RV failure from animal models and human RHF.^193^^,^^194^ This approach helps to identify the specific genes and proteins abnormally expressed in RHD, to help elaborate specific and adapted treatments for each patient.

In this context, clinical trials are ongoing, aiming to treat ARVC in a genotype-specific manner, particularly focusing on gene therapy, which represents a shift toward precision medicine in ARVC care. *PKP2* gene variants are the most common genetic cause of ARVC.^195^ Hence, *PKP2* is a target for genotype-specific therapies. In a phase 1b clinical trial called RIDGE-1 (NCT06228924), the safety, tolerability, and efficacy of TN-401 (an AAV-based gene therapy designed to deliver a functional *PKP2* gene) are evaluated in adult patients with ARVC caused by PKP2 mutations.^196^ In a phase 1/2 study called HEROIC-PKP (NCT06109181), the role of the delivery of a functional *PKP2* gene to cardiac cells is evaluated in adults with confirmed *PKP2* pathogenic variants.^197^

Personalized medicine strategies remain to be consolidated and improved, but this approach carries undeniable potential to positively impact outcomes (Table 2).

### Perspectives on the implications of artificial intelligence (diagnosis and treatment)

The utilization of artificial intelligence (AI) has shown efficacy in predicting the development of RHD, and RHD risk factors.^198^^,^^199^ As one example, AI models have shown a promising level of discrimination in identifying patients who develop RV failure post implantation of a LV assist device.^200^

The potential of AI to provide the appropriate diagnosis and treatment option in various cardiac disorders including RHD is an interesting avenue that could potentially combine existing and new therapeutic approaches with personalized medicine.^198^^,^^200^

Utilization of AI and machine learning in echocardiography and cardiovascular magnetic resonance have shown efficacy in improving image acquisition, image analysis, diagnosis, and prognosis.^201^^,^^202^ In a retrospective study involving 31,752 patients, a machine learning score using cardiovascular magnetic resonance data had greater prognostic ability in predicting 10-year death, compared with traditional clinical or cardiovascular magnetic resonance scores.^203^ In the context of RHD, AI algorithms offer exceptional ability and the potential to combine information obtained from echocardiography, cardiovascular magnetic resonance, medical records, and genetic information, to provide a more detailed vision of the patient’s condition, a more personalized diagnosis, and more precise prediction of the risk of arrhythmias^204^ (Table 2). More investigations are required to improve the use of AI in RHD diagnosis and management.

## Conclusions

Patients with RHD are susceptible to cardiac arrhythmias, including AF, atrial flutter, ventricular arrhythmias, and sudden death. Although important advances have been made in understanding arrhythmogenesis in RHD, further studies are necessary to clarify the underlying mechanisms. Chronic and unresolved inflammation has emerged as an important contributor to myocardial fibrosis and arrhythmias in RHD (Central Illustration). New therapeutic strategies might involve anti-inflammation approaches to prevent and treat RHD-induced inflammation and cardiac arrhythmias.

**Central Illustration:**
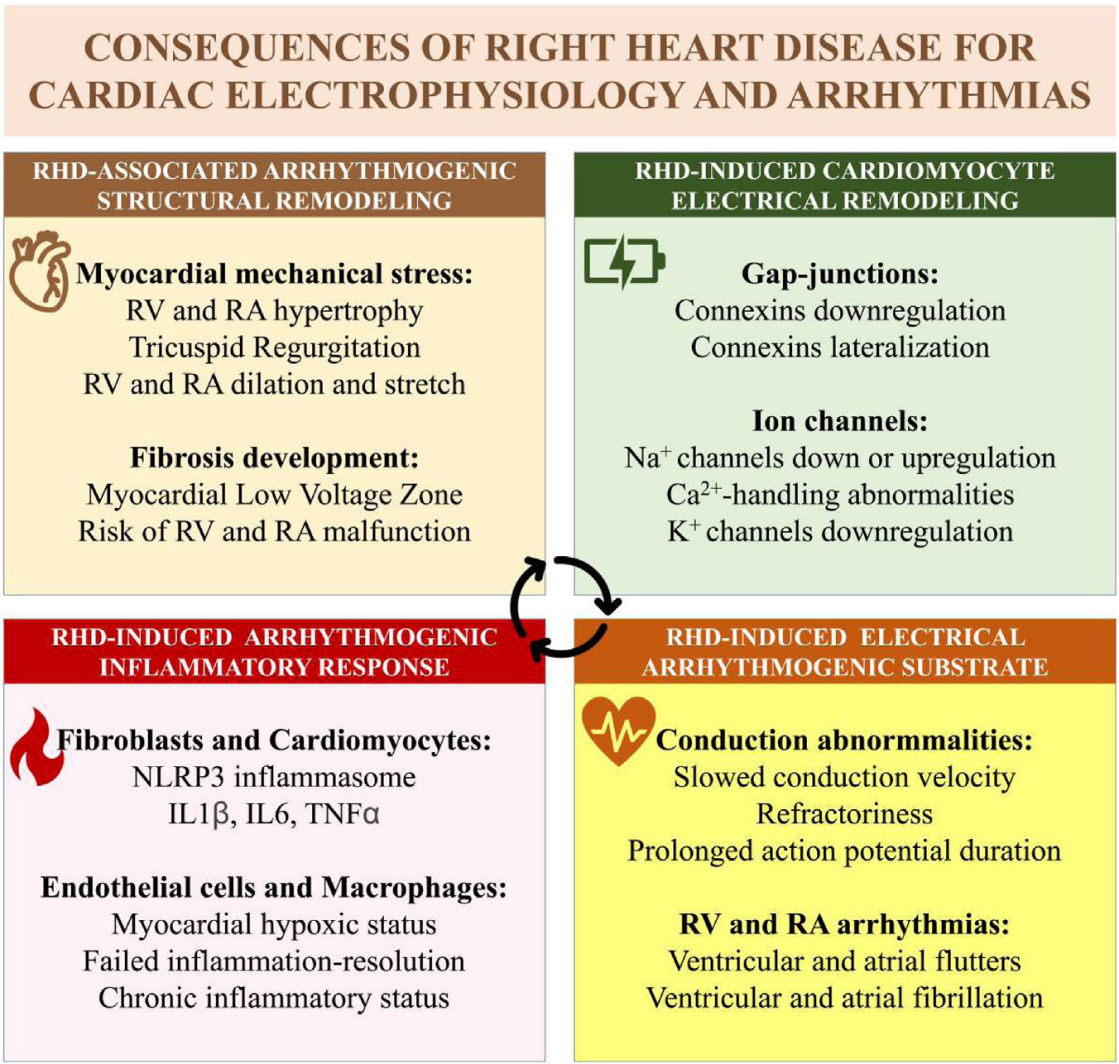
Consequences of right-heart disease (RHD) for cardiac electrophysiology and arrhythmias. In RHD, arrhythmogenic remodelling, including structural and functional changes accompanied by myocardial inflammation and the perturbation of cardiac electrical conduction, lead to atrial and/or ventricular arrhythmias, including ventricular and atrial fibrillation. Ca^2+^, calcium; IL, interleukin; K^+^, potassium; Na^+^, sodium; NLRP3, nucleotide-binding domain, leucine-rich-containing family, pyrin domain-containing-3 inflammasome; RA, right atrial; RV, right ventricular; TNF-α, tumour necrosis factor alpha.

## References

[1] Kret M., Arora R. (2007). Pathophysiological basis of right ventricular remodeling. J Cardiovasc Pharmacol Ther.

[2] Rako Z.A., Kremer N., Yogeswaran A., Richter M.J., Tello K. (2023). Adaptive versus maladaptive right ventricular remodelling. ESC Heart Fail.

[3] Dini F.L., Pugliese N.R., Ameri P. (2023). Right ventricular failure in left heart disease: from pathophysiology to clinical manifestations and prognosis. Heart Fail Rev.

[4] Al-Omary M.S., Sugito S., Boyle A.J., Sverdlov A.L., Collins N.J. (2020). Pulmonary hypertension due to left heart disease: diagnosis, pathophysiology, and therapy. Hypertension.

[5] Kolb T.M., Hassoun P.M. (2012). Right ventricular dysfunction in chronic lung disease. Cardiol Clin.

[6] Shah P.H., Lee J.H., Salvi D.J. (2021). Cardiovascular system involvement in cystic fibrosis. Cureus.

[7] Arrigo M., Huber L.C. (2021). Pulmonary embolism and heart failure: a reappraisal. Card Fail Rev.

[8] Cassady S.J., Ramani G.V. (2020). Right heart failure in pulmonary hypertension. Cardiol Clin.

[9] Bruce C.J., Connolly H.M. (2009). Right-sided valve disease deserves a little more respect. Circulation.

[10] Ávila P., Mercier L.A., Dore A. (2014). Adult congenital heart disease: a growing epidemic. Can J Cardiol.

[11] van der Bruggen C.E.E., Tedford R.J., Handoko M.L., van der Velden J., de Man F.S. (2017). RV pressure overload: from hypertrophy to failure. Cardiovasc Res.

[12] O'Meara K., Stone G., Buch E. (2024). Atrial arrhythmias in patients with pulmonary hypertension. Chest.

[13] Hiram R., Provencher S. (2021). Pulmonary disease, pulmonary hypertension and atrial fibrillation. Card Electrophysiol Clin.

[14] Umar S., Lee J.H., de Lange E. (2012). Spontaneous ventricular fibrillation in right ventricular failure secondary to chronic pulmonary hypertension. Circ Arrhythm Electrophysiol.

[15] Bessiere F., Mondesert B., Chaix M.A., Khairy P. (2021). Arrhythmias in adults with congenital heart disease and heart failure. Heart Rhythm O2.

[16] Wanamaker B., Cascino T., McLaughlin V. (2018). Atrial arrhythmias in pulmonary hypertension: pathogenesis, prognosis and management. Arrhythm Electrophysiol Rev.

[17] Bernal-Ramirez J., Díaz-Vesga M.C., Talamilla M. (2021). Exploring functional differences between the right and left ventricles to better understand right ventricular dysfunction. Oxid Med Cell Longev.

[18] Triposkiadis F., Xanthopoulos A., Boudoulas K.D. (2022). The interventricular septum: structure, function, dysfunction, and diseases. J Clin Med.

[19] Haddad F., Hunt S.A., Rosenthal D.N., Murphy D.J. (2008). Right ventricular function in cardiovascular disease, part I: anatomy, physiology, aging, and functional assessment of the right ventricle. Circulation.

[20] Hartupee J., Mann D.L. (2017). Neurohormonal activation in heart failure with reduced ejection fraction. Nat Rev Cardiol.

[21] Manolis A.A., Manolis T.A., Manolis A.S. (2023). Neurohumoral activation in heart failure. Int J Mol Sci.

[22] Eisenberg E., Di Palo K.E., Piña I.L. (2018). Sex differences in heart failure. Clin Cardiol.

[23] DeFilippis E.M., Beale A., Martyn T. (2022). Heart failure subtypes and cardiomyopathies in women. Circ Res.

[24] Regitz-Zagrosek V. (2020). Sex and gender differences in heart failure. Int J Heart Fail.

[25] Rosenkranz S., Gibbs J.S., Wachter R. (2016). Left ventricular heart failure and pulmonary hypertension. Eur Heart J.

[26] Farber H.W., Gibbs S. (2015). Under pressure: pulmonary hypertension associated with left heart disease. Eur Respir Rev.

[27] Thandavarayan R.A., Chitturi K.R., Guha A. (2020). Pathophysiology of acute and chronic right heart failure. Cardiol Clin.

[28] Leopold J.A., Kawut S.M., Aldred M.A. (2021). Diagnosis and treatment of right heart failure in pulmonary vascular diseases: a National Heart, Lung, and Blood Institute workshop. Circ Heart Fail.

[29] Mendiola E.A., da Silva Gonçalves Bos D., Leichter D.M. (2023). Right ventricular architectural remodeling and functional adaptation in pulmonary hypertension. Circ Heart Fail.

[30] Konstam M.A., Kiernan M.S., Bernstein D. (2018). Evaluation and management of right-sided heart failure: a scientific statement from the American Heart Association. Circulation.

[31] Jankowich M., Abbasi S.A., Vang A., Choudhary G. (2019). Right ventricular fibrosis is related to pulmonary artery stiffness in pulmonary hypertension: a cardiac magnetic resonance imaging study. Am J Respir Crit Care Med.

[32] Rodriguez-Arias J.J., García-Álvarez A. (2021). Sex differences in pulmonary hypertension. Front Aging.

[33] Melenovsky V., Hwang S.J., Lin G., Redfield M.M., Borlaug B.A. (2014). Right heart dysfunction in heart failure with preserved ejection fraction. Eur Heart J.

[34] Imran T.F., Ataklte F., Khalid M. (2024). Clinical predictors of right ventricular dysfunction and association with adverse outcomes in peripartum cardiomyopathy. ESC Heart Fail.

[35] Mertens L., Khairy P. (2013). Right ventricular diastolic function in congenital heart disease. Can J Cardiol.

[36] Dahou A., Levin D., Reisman M., Hahn R.T. (2019). Anatomy and physiology of the tricuspid valve. JACC Cardiovasc Imaging.

[37] Coffey S., Rayner J., Newton J., Prendergast B.D. (2014). Right-sided valve disease. Int J Clin Pract.

[38] Nemoto N., Lesser J.R., Pedersen W.R. (2015). Pathogenic structural heart changes in early tricuspid regurgitation. J Thorac Cardiovasc Surg.

[39] Marshall W.H.V., Daniels C.J. (2024). Progression of valvular pulmonic stenosis in adulthood: Never say never. CASE (Phila).

[40] Ryan J.J., Archer S.L. (2014). The right ventricle in pulmonary arterial hypertension: disorders of metabolism, angiogenesis and adrenergic signaling in right ventricular failure. Circ Res.

[41] Pascall E., Tulloh R.M. (2018). Pulmonary hypertension in congenital heart disease. Future Cardiol.

[42] Lozek M., Kovanda J., Kubus P. (2024). How to assess and treat right ventricular electromechanical dyssynchrony in post-repair tetralogy of Fallot: insights from imaging, invasive studies, and computational modelling. Europace.

[43] Arshad H.B., Duarte V.E. (2021). Evaluation and management of pulmonary arterial hypertension in congenital heart disease. Methodist Debakey Cardiovasc J.

[44] Parodi O., Neglia D., Marcassa C., Marzullo P., Sambuceti G. (1991). Right coronary artery disease. Pathophysiology, clinical relevance, and methods for recognition. Circulation.

[45] Sen G., Veitch A., Sinha M. (2020). Hyper-dominance of the left anterior descending artery—a large territory. BJR Case Rep.

[46] Ballesteros L.E., Ramirez L.M., Quintero I.D. (2011). Right coronary artery anatomy: anatomical and morphometric analysis. Rev Bras Cir Cardiovasc.

[47] Wu B., Kheiwa A., Swamy P. (2024). Clinical significance of coronary arterial dominance: a review of the literature. J Am Heart Assoc.

[48] Shriki J.E., Shinbane J.S., Rashid M.A. (2012). Identifying, characterizing, and classifying congenital anomalies of the coronary arteries. Radiographics.

[49] Hamaguchi K., Sakakura K., Jinnouchi H. (2025). Comparison of clinical outcomes between proximal and non-proximal right coronary artery occlusion in patients with inferior ST-segment elevation myocardial infarction. J Cardiol.

[50] Kanza R.E., Ayoub S., Bonenfant F. (2020). Incidental, non-gated thoracic CT angiographic detection of proximal right coronary artery total occlusion associated with acute myocardial infarction. Eur J Radiol Open.

[51] Vives-Borrás M., Maestro A., García-Hernando V. (2019). Electrocardiographic distinction of left circumflexand right coronary artery occlusion in patientswith inferior acute myocardial infarction. Am J Cardiol.

[52] Gregg R.E., Fiol-Sala M., Nikus K.C. (2012). Automated discrimination of proximal right coronary artery occlusion from middle-to-distal right coronary artery occlusion and left circumflex occlusion in ST-elevation myocardial infarction. J Electrocardiol.

[53] Bairey C.N., Shah P.K., Lew A.S., Hulse S. (1987). Electrocardiographic differentiation of occlusion of the left circumflex versus the right coronary artery as a cause of inferior acute myocardial infarction. Am J Cardiol.

[54] Huey B.L., Beller G.A., Kaiser D.L., Gibson R.S. (1988). A comprehensive analysis of myocardial infarction due to left circumflex artery occlusion: comparison with infarction due to right coronary artery and left anterior descending artery occlusion. J Am Coll Cardiol.

[55] Femia G., French J.K., Juergens C., Leung D., Lo S. (2021). Right ventricular myocardial infarction: pathophysiology, clinical implications and management. Rev Cardiovasc Med.

[56] Pinsky M.R. (2016). The right ventricle: interaction with the pulmonary circulation. Crit Care.

[57] Gorter T.M., Lexis C.P., Hummel Y.M. (2016). Right ventricular function after acute myocardial infarction treated with primary percutaneous coronary intervention (from the Glycometabolic Intervention as Adjunct to Primary Percutaneous Coronary Intervention in ST-Segment Elevation Myocardial Infarction III Trial). Am J Cardiol.

[58] Shakir D.K., Arafa S.O. (2007). Right atrial infarction, atrial arrhythmia and inferior myocardial infarction form a missed triad: a case report and review of the literature. Can J Cardiol.

[59] Kakizaki R., Imaki R., Ako J. (2023). Right atrial infarction causing recurrent cardiac rupture and cardiac tamponade. Eur Heart J Case Rep.

[60] Hurley R.W., Subramanian R., Rahko P.S., Shelp W.D. (1990). Isolated right atrial infarction with rupture. N Engl J Med.

[61] Ondrus T., Kanovsky J., Novotny T. (2013). Right ventricular myocardial infarction: from pathophysiology to prognosis. Exp Clin Cardiol.

[62] Kawada S., Chakraborty P., Kakarla J. (2025). Role of subpulmonary right ventricle in sudden cardiac death in adults with congenital heart disease. Heart Rhythm.

[63] Beyls C., Hermida A., Martin N. (2024). Prognostic value of right ventricular longitudinal shortening fraction in patients with ST-elevation myocardial infarction: a prospective echocardiography study. Am J Cardiol.

[64] Majos E., Dąbrowski R., Szwed H. (2013). The right ventricle in patients with chronic heart failure and atrial fibrillation. Cardiol J.

[65] Fang Y.H., Piao L., Hong Z. (2012). Therapeutic inhibition of fatty acid oxidation in right ventricular hypertrophy: exploiting Randle's cycle. J Mol Med (Berl).

[66] Fürniss H.E., Wülfers E.M., Iaconianni P. (2024). Disease severity, arrhythmogenesis, and fibrosis are related to longer action potentials in tetralogy of Fallot. Clin Res Cardiol.

[67] Gorter T.M., van Melle J.P., Rienstra M. (2018). Right heart dysfunction in heart failure with preserved ejection fraction: the impact of atrial fibrillation. J Card Fail.

[68] Arvidsson S., Henein M.Y., Wikström G., Suhr O.B., Lindqvist P. (2018). Right ventricular involvement in transthyretin amyloidosis. Amyloid.

[69] Garcia-Pavia P., Rapezzi C., Adler Y. (2021). Diagnosis and treatment of cardiac amyloidosis: a position statement of the ESC Working Group on Myocardial and Pericardial Diseases. Eur Heart J.

[70] Patel R.K., Ioannou A., Razvi Y. (2022). Sex differences among patients with transthyretin amyloid cardiomyopathy—from diagnosis to prognosis. Eur J Heart Fail.

[71] Keteepe-Arachi T., Sharma S. (2017). Cardiovascular disease in women: understanding symptoms and risk factors. Eur Cardiol.

[72] Shah K.B., Inoue Y., Mehra M.R. (2006). Amyloidosis and the heart: a comprehensive review. Arch Intern Med.

[73] Harms H.J., Clemmensen T., Rosengren S. (2023). Association of right ventricular myocardial blood flow with pulmonary pressures and outcome in cardiac amyloidosis. JACC Cardiovasc Imaging.

[74] Imdad U. (2023). Amyloidosis of the heart: a comprehensive review. Cureus.

[75] Vergaro G., Aimo A., Rapezzi C. (2022). Atrial amyloidosis: mechanisms and clinical manifestations. Eur J Heart Fail.

[76] Roche S.L., Redington A.N. (2013). The failing right ventricle in congenital heart disease. Can J Cardiol.

[77] Rojas S.F., Nattel S., Hiram R., Khairy P. (2025). Right ventricular electrophysiology and arrhythmias in adults with congenital heart disease: scientific basis for management. Can J Cardiol.

[78] Martín de Miguel I., Ávila P. (2021). Atrial fibrillation in congenital heart disease. Eur Cardiol.

[79] Marcus F.I., Edson S., Towbin J.A. (2013). Genetics of arrhythmogenic right ventricular cardiomyopathy: a practical guide for physicians. J Am Coll Cardiol.

[80] Choudhary N., Tompkins C., Polonsky B. (2016). Clinical presentation and outcomes by sex in arrhythmogenic right ventricular cardiomyopathy: findings from the North American ARVC Registry. J Cardiovasc Electrophysiol.

[81] Corrado D., Thiene G. (2006). Arrhythmogenic right ventricular cardiomyopathy/dysplasia: clinical impact of molecular genetic studies. Circulation.

[82] Krahn A.D., Wilde A.A.M., Calkins H. (2022). Arrhythmogenic right ventricular cardiomyopathy. JACC Clin Electrophysiol.

[83] James C.A., Jongbloed J.D.H., Hershberger R.E. (2021). International evidence based reappraisal of genes associated with arrhythmogenic right ventricular cardiomyopathy using the clinical genome resource framework. Circ Genom Precis Med.

[84] Garcia-Montero M., Fanous Y., Krahn A.D. (2025). New insights into genetic right ventricular cardiomyopathies. Can J Cardiol.

[85] Li K.H.C., Bazoukis G., Liu T. (2017). Arrhythmogenic right ventricular cardiomyopathy/dysplasia (ARVC/D) in clinical practice. J Arrhythm.

[86] Cadrin-Tourigny J., Bosman L.P., Wang W. (2021). sudden cardiac death prediction in arrhythmogenic right ventricular cardiomyopathy: a multinational collaboration. Circ Arrhythm Electrophysiol.

[87] Baturova M.A., Haugaa K.H., Jensen H.K. (2020). Atrial fibrillation as a clinical characteristic of arrhythmogenic right ventricular cardiomyopathy: experience from the Nordic ARVC Registry. Int J Cardiol.

[88] Alhassani S., Deif B., Conacher S., Cunningham K.S., Roberts J.D. (2018). A large familial pathogenic Plakophilin-2 gene (PKP2) deletion manifesting with sudden cardiac death and lone atrial fibrillation: evidence for alternating atrial and ventricular phenotypes. HeartRhythm Case Rep.

[89] Jordà P., Bosman L.P., Gasperetti A. (2022). Arrhythmic risk prediction in arrhythmogenic right ventricular cardiomyopathy: external validation of the arrhythmogenic right ventricular cardiomyopathy risk calculator. Eur Heart J.

[90] Cadrin-Tourigny J., Bosman L.P., Nozza A. (2019). A new prediction model for ventricular arrhythmias in arrhythmogenic right ventricular cardiomyopathy. Eur Heart J.

[91] Li K.H.C., Lee S., Yin C. (2020). Brugada syndrome: a comprehensive review of pathophysiological mechanisms and risk stratification strategies. Int J Cardiol Heart Vasc.

[92] Liantonio A., Bertini M., Mele A. (2023). Brugada Syndrome: more than a monogenic channelopathy. Biomedicines.

[93] Batchvarov V.N. (2014). The Brugada Syndrome—diagnosis, clinical implications and risk stratification. Eur Cardiol.

[94] Khairy P., Aboulhosn J., Gurvitz M.Z. (2010). Arrhythmia burden in adults with surgically repaired tetralogy of Fallot: a multi-institutional study. Circulation.

[95] Lundqvist C.B., Potpara T.S., Malmborg H. (2017). Supraventricular arrhythmias in patients with adult congenital heart disease. Arrhythm Electrophysiol Rev.

[96] Corrado D., Zorzi A., Cerrone M. (2016). Relationship between arrhythmogenic right ventricular cardiomyopathy and Brugada syndrome: new insights from molecular biology and clinical implications. Circ Arrhythm Electrophysiol.

[97] Kelly A., Salerno S., Connolly A. (2018). Normal interventricular differences in tissue architecture underlie right ventricular susceptibility to conduction abnormalities in a mouse model of Brugada syndrome. Cardiovasc Res.

[98] Behr E.R., Ben-Haim Y., Ackerman M.J., Krahn A.D., Wilde A.A.M. (2021). Brugada syndrome and reduced right ventricular outflow tract conduction reserve: a final common pathway?. Eur Heart J.

[99] Steiner R., Makarovic S., Makarovic Z., Bilic-Curcic I. (2014). Brugada syndrome and right ventricle morphofunctional abnormalities on echocardiography in young male with family anamnesis of sudden cardiac death. Coll Antropol.

[100] Sharifi Kia D., Kim K., Simon M.A. (2021). Current understanding of the right ventricle structure and function in pulmonary arterial hypertension. Front Physiol.

[101] Zhou Z., Huang Y., Han L. (2023). Right ventricular dilatation score: a new assessment to right ventricular dilatation in adult patients with repaired tetralogy of Fallot. BMC Cardiovasc Disord.

[102] Medvedev R.Y., Afolabi S.O., Turner D.G.P., Glukhov A.V. (2024). Mechanisms of stretch-induced electro-anatomical remodeling and atrial arrhythmogenesis. J Mol Cell Cardiol.

[103] Thomas T.P., Grisanti L.A. (2020). The dynamic interplay between cardiac inflammation and fibrosis. Front Physiol.

[104] Cai K., Jiang H., Zou Y. (2024). Programmed death of cardiomyocytes in cardiovascular disease and new therapeutic approaches. Pharmacol Res.

[105] Ammirati E., Frigerio M., Adler E.D. (2020). Management of acute myocarditis and chronic inflammatory cardiomyopathy: an expert consensus document. Circ Heart Fail.

[106] Al-Kaisey A.M., Parameswaran R., Joseph S.A. (2021). Extensive right atrial free wall low-voltage zone as the substrate for atrial fibrillation: successful ablation by scar homogenization. Europace.

[107] Hiram R. (2021). Cardiac cytokine therapy? Relevance of targeting inflammatory mediators to combat cardiac arrhythmogenic remodeling. Int J Cardiol Heart Vasc.

[108] Hammarström E., Wranne B., Pinto F.J., Puryear J., Popp R.L. (1991). Tricuspid annular motion. J Am Soc Echocardiogr.

[109] Modin D., Møgelvang R., Andersen D.M., Biering-Sørensen T. (2019). Right ventricular function evaluated by tricuspid annular plane systolic excursion predicts cardiovascular death in the general population. J Am Heart Assoc.

[110] Ko K.Y., Jang J.H., Choi S.H. (2022). Impact of right atrial enlargement on clinical outcome in patients with atrial fibrillation. Front Cardiovasc Med.

[111] Wu Q.Q., Xiao Y., Yuan Y. (2017). Mechanisms contributing to cardiac remodelling. Clin Sci (Lond).

[112] Hayward R.M., Tseng Z.H. (2014). Arrhythmias in complex congenital heart disease. Card Electrophysiol Clin.

[113] Le Quilliec E., LeBlanc C.A., Neuilly O. (2024). Atrial cardiomyocytes contribute to the inflammatory status associated with atrial fibrillation in right heart disease. Europace.

[114] Purcell N.H., Tang G., Yu C. (2001). Activation of NF-kappa B is required for hypertrophic growth of primary rat neonatal ventricular cardiomyocytes. Proc Natl Acad Sci U S A.

[115] Liu T., Zhang L., Joo D., Sun S.C. (2017). NF-kappa B signaling in inflammation. Signal Transduct Target Ther.

[116] Lindner D., Zietsch C., Tank J. (2014). Cardiac fibroblasts support cardiac inflammation in heart failure. Basic Res Cardiol.

[117] Giannandrea M., Parks W.C. (2014). Diverse functions of matrix metalloproteinases during fibrosis. Dis Model Mech.

[118] Younes R., LeBlanc C.A., Hiram R. (2022). Evidence of failed resolution mechanisms in arrhythmogenic inflammation, fibrosis and right heart disease. Biomolecules.

[119] Frangogiannis N.G. (2017). Fibroblasts and the extracellular matrix in right ventricular disease. Cardiovasc Res.

[120] Chong A.Y., Blann A.D., Patel J. (2004). Endothelial dysfunction and damage in congestive heart failure: relation of flow-mediated dilation to circulating endothelial cells, plasma indexes of endothelial damage, and brain natriuretic peptide. Circulation.

[121] Fang Y., Wu D., Birukov K.G. (2019). Mechanosensing and mechanoregulation of endothelial cell functions. Compr Physiol.

[122] Kume O., Teshima Y., Abe I. (2017). Role of atrial endothelial cells in the development of atrial fibrosis and fibrillation in response to pressure overload. Cardiovasc Pathol.

[123] Ullah K., Wu R. (2021). Hypoxia-inducible factor regulates endothelial metabolism in cardiovascular disease. Front Physiol.

[124] Kassa B., Kumar R., Mickael C. (2021). Endothelial cell PHD2-HIF1alpha-PFKFB3 contributes to right ventricle vascular adaptation in pulmonary hypertension. Am J Physiol Lung Cell Mol Physiol.

[125] Shahid F., Lip G.Y.H., Shantsila E. (2018). Role of monocytes in heart failure and atrial fibrillation. J Am Heart Assoc.

[126] Al-Qazazi R., Lima P.D.A., Prisco S.Z. (2022). Macrophage-NLRP3 activation promotes right ventricle failure in pulmonary arterial hypertension. Am J Respir Crit Care Med.

[127] Hiram R., Xiong F., Naud P. (2024). An inflammation resolution-promoting intervention prevents atrial fibrillation caused by left ventricular dysfunction. Cardiovasc Res.

[128] Willerson J.T., Ridker P.M. (2004). Inflammation as a cardiovascular risk factor. Circulation.

[129] Hiram R. (2024). The duration of atrial fibrillation might be associated with right heart disease severity. Int J Cardiol.

[130] Hiram R. (2022). Resolution-promoting autacoids demonstrate promising cardioprotective effects against heart diseases. Mol Biol Rep.

[131] Dagher L., Shi H., Zhao Y. (2021). Atrial fibrosis progression in patients with no history of atrial fibrillation. J Cardiovasc Electrophysiol.

[132] Platonov P.G. (2017). Atrial fibrosis: an obligatory component of arrhythmia mechanisms in atrial fibrillation?. J Geriatr Cardiol.

[133] Lei M., Salvage S.C., Jackson A.P., Huang C.L. (2024). Cardiac arrhythmogenesis: roles of ion channels and their functional modification. Front Physiol.

[134] Siebermair J., Suksaranjit P., McGann C.J. (2019). Atrial fibrosis in non-atrial fibrillation individuals and prediction of atrial fibrillation by use of late gadolinium enhancement magnetic resonance imaging. J Cardiovasc Electrophysiol.

[135] Mikhael M., Makar C., Wissa A. (2019). Oxidative stress and its implications in the right ventricular remodeling secondary to pulmonary hypertension. Front Physiol.

[136] Bove T., Alipour Symakani R., Verbeke J. (2020). Study of the time-relationship of the mechano-electrical interaction in an animal model of tetralogy of Fallot: implications for the risk assessment of ventricular arrhythmias. Interact Cardiovasc Thorac Surg.

[137] Asimaki A., Saffitz J.E. (2012). Gap junctions and arrhythmogenic cardiomyopathy. Heart Rhythm.

[138] Salameh A., Blanke K., Daehnert I. (2013). Role of connexins in human congenital heart disease: the chicken and egg problem. Front Pharmacol.

[139] Lee J.K., Nishiyama A., Kambe F. (1999). Downregulation of voltage-gated K(+) channels in rat heart with right ventricular hypertrophy. Am J Physiol.

[140] Diness J.G., Bentzen B.H., Sørensen U.S., Grunnet M. (2015). Role of calcium-activated potassium channels in atrial fibrillation pathophysiology and therapy. J Cardiovasc Pharmacol.

[141] Lambert M., Boet A., Rucker-Martin C. (2018). Loss of KCNK3 is a hallmark of RV hypertrophy/dysfunction associated with pulmonary hypertension. Cardiovasc Res.

[142] Benoist D., Dubes V., Roubertie F. (2017). Proarrhythmic remodelling of the right ventricle in a porcine model of repaired tetralogy of Fallot. Heart.

[143] Song W., Shou W. (2012). Cardiac sodium channel Nav1.5 mutations and cardiac arrhythmia. Pediatr Cardiol.

[144] Chiu S.N., Tsai C.T., Lin L.Y. (2015). Repolarization alternans and ventricular arrhythmia in a repaired tetralogy of Fallot animal model. J Am Heart Assoc.

[145] Cho G.W., Altamirano F., Hill J.A. (2016). Chronic heart failure: Ca(2+), catabolism, and catastrophic cell death. Biochim Biophys Acta.

[146] Sabourin J., Boet A., Rucker-Martin C. (2018). Ca2+ handling remodeling and STIM1L/Orai1/TRPC1/TRPC4 upregulation in monocrotaline-induced right ventricular hypertrophy. J Mol Cell Cardiol.

[147] Benoist D., Stones R., Drinkhill M.J. (2012). Cardiac arrhythmia mechanisms in rats with heart failure induced by pulmonary hypertension. Am J Physiol Heart Circ Physiol.

[148] Remme C.A. (2013). Cardiac sodium channelopathy associated with SCN5A mutations: electrophysiological, molecular and genetic aspects. J Physiol.

[149] Schaffer P., Pelzmann B., Bernhart E. (1999). Repolarizing currents in ventricular myocytes from young patients with tetralogy of Fallot. Cardiovasc Res.

[150] Saura M., Zamorano J.L., Zaragoza C. (2022). Preclinical models of congestive heart failure, advantages, and limitations for application in clinical practice. Front Physiol.

[151] Andersen A., van der Feen D.E., Andersen S. (2020). Animal models of right heart failure. Cardiovasc Diagn Ther.

[152] Ebert A.D., Liang P., Wu J.C. (2012). Induced pluripotent stem cells as a disease modeling and drug screening platform. J Cardiovasc Pharmacol.

[153] de la Roche J., Angsutararux P., Kempf H. (2019). Comparing human iPSC-cardiomyocytes versus HEK293T cells unveils disease-causing effects of Brugada mutation A735V of NaV1.5 sodium channels. Sci Rep.

[154] Pérez-Hernández M., Matamoros M., Alfayate S. (2018). Brugada syndrome trafficking-defective Nav1.5 channels can trap cardiac Kir2.1/2.2 channels. JCI Insight.

[155] Inoue H., Nakamura S., Higo S. (2022). Modeling reduced contractility and impaired desmosome assembly due to plakophilin-2 deficiency using isogenic iPS cell-derived cardiomyocytes. Stem Cell Rep.

[156] Li W., Stauske M., Luo X. (2020). Disease phenotypes and mechanisms of iPSC-derived cardiomyocytes from Brugada syndrome patients with a loss-of-function SCN5A mutation. Front Cell Dev Biol.

[157] Nijak A., Saenen J., Labro A.J. (2021). iPSC-cardiomyocyte models of Brugada syndrome-achievements, challenges and future perspectives. Int J Mol Sci.

[158] Smith J.G.W., Owen T., Bhagwan J.R. (2018). Isogenic pairs of hiPSC-CMs with hypertrophic cardiomyopathy/LVNC-associated ACTC1 E99K mutation unveil differential functional deficits. Stem Cell Rep.

[159] Sun Y., Su J., Wang X. (2023). Patient-specific iPSC-derived cardiomyocytes reveal variable phenotypic severity of Brugada syndrome. EBioMedicine.

[160] Karakikes I., Stillitano F., Nonnenmacher M. (2015). Correction of human phospholamban R14del mutation associated with cardiomyopathy using targeted nucleases and combination therapy. Nat Commun.

[161] Joshi J., Albers C., Smole N., Guo S., Smith S.A. (2024). Human induced pluripotent stem cell-derived cardiomyocytes (iPSC-CMs) for modeling cardiac arrhythmias: strengths, challenges and potential solutions. Front Physiol.

[162] Tzatzalos E., Abilez O.J., Shukla P., Wu J.C. (2016). Engineered heart tissues and induced pluripotent stem cells: macro- and microstructures for disease modeling, drug screening, and translational studies. Adv Drug Deliv Rev.

[163] Bueno-Beti C., Sassi Y., Hajjar R.J., Hadri L. (2018). Pulmonary artery hypertension model in rats by monocrotaline administration. Methods Mol Biol.

[164] Krstic A.M., Jones T.L.M., Power A.S., Ward M.L. (2014). The monocrotaline rat model of right heart disease induced by pulmonary artery hypertension. Biomedicines.

[165] Hiram R., Xiong F., Naud P. (2021). The inflammation-resolution promoting molecule resolvin-D1 prevents atrial proarrhythmic remodelling in experimental right heart disease. Cardiovasc Res.

[166] Wu J., Liu T., Shi S. (2022). Dapagliflozin reduces the vulnerability of rats with pulmonary arterial hypertension-induced right heart failure to ventricular arrhythmia by restoring calcium handling. Cardiovasc Diabetol.

[167] LeBlanc C.A., Higgins M.È., Sirois M.G. (2024). Rat model of right-sided cardiac remodeling and arrhythmia using pulmonary artery banding. J Vis Exp.

[168] Sicard P., Jouitteau T., Andrade-Martins T. (2019). Right coronary artery ligation in mice: a novel method to investigate right ventricular dysfunction and biventricular interaction. Am J Physiol Heart Circ Physiol.

[169] Nishida K., Qi X.Y., Wakili R. (2011). Mechanisms of atrial tachyarrhythmias associated with coronary artery occlusion in a chronic canine model. Circulation.

[170] Johny E., Dutta P. (2022). Left coronary artery ligation: a surgical murine model of myocardial infarction. J Vis Exp.

[171] Zhang P., Da Silva Goncalves Bos D., Vang A. (2024). Reduced exercise capacity occurs before intrinsic skeletal muscle dysfunction in experimental rat models of pulmonary hypertension. Pulm Circ.

[172] Bikou O., Sassi Y. (2024). The Sugen/hypoxia rat model for pulmonary hypertension and right heart failure. Methods Mol Biol.

[173] Golob M.J., Wang Z., Prostrollo A.J., Hacker T.A., Chesler N.C. (2016). Limiting collagen turnover via collagenase-resistance attenuates right ventricular dysfunction and fibrosis in pulmonary arterial hypertension. Physiol Rep.

[174] Martin B., Vanderpool R.R., Henry B.L. (2021). Relaxin inhibits ventricular arrhythmia and asystole in rats with pulmonary arterial hypertension. Front Cardiovasc Med.

[175] Engelhardt S., Hein L., Wiesmann F., Lohse M.J. (1999). Progressive hypertrophy and heart failure in beta1-adrenergic receptor transgenic mice. Proc Natl Acad Sci U S A.

[176] Broman M.T., Nadadur R.D., Perez-Cervantes C. (2024). A genomic link from heart failure to atrial fibrillation risk: FOG2 modulates a TBX5/GATA4-dependent atrial gene regulatory network. Circulation.

[177] Fujita M., Mason R.J., Cool C. (2002). Pulmonary hypertension in TNF-alpha-overexpressing mice is associated with decreased VEGF gene expression. J Appl Physiol (1985).

[178] Haudek S.B., Taffet G.E., Schneider M.D., Mann D.L. (2007). TNF provokes cardiomyocyte apoptosis and cardiac remodeling through activation of multiple cell death pathways. J Clin Invest.

[179] Saba S., Janczewski A.M., Baker L.C. (2005). Atrial contractile dysfunction, fibrosis, and arrhythmias in a mouse model of cardiomyopathy secondary to cardiac-specific overexpression of tumor necrosis factor-{alpha. Am J Physiol Heart Circ Physiol.

[180] Wu I., Zeng A., Greer-Short A. (2024). AAV9:PKP2 improves heart function and survival in a Pkp2-deficient mouse model of arrhythmogenic right ventricular cardiomyopathy. Commun Med (Lond).

[181] Gerull B., Brodehl A. (2020). Genetic animal models for arrhythmogenic cardiomyopathy. Front Physiol.

[182] Tester D.J., Ackerman J.P., Giudicessi J.R. (2019). Plakophilin-2 truncation variants in patients clinically diagnosed with catecholaminergic polymorphic ventricular tachycardia and decedents with exercise-associated autopsy negative sudden unexplained death in the young. JACC Clin Electrophysiol.

[183] Sendfeld F., Selga E., Scornik F.S. (2019). Experimental models of Brugada syndrome. Int J Mol Sci.

[184] Xu W.J., Wu Q., He W.N. (2023). Interleukin-6 and pulmonary hypertension: from physiopathology to therapy. Front Immunol.

[185] Prins K.W., Archer S.L., Pritzker M. (2018). Interleukin-6 is independently associated with right ventricular function in pulmonary arterial hypertension. J Heart Lung Transplant.

[186] Mundisugih J., Ravindran D., Kizana E. (2024). Exploring the therapeutic potential of gene therapy in arrhythmogenic right ventricular cardiomyopathy. Biomedicines.

[187] Rai N., Shihan M., Seeger W., Schermuly R.T., Novoyatleva T. (2021). Genetic delivery and gene therapy in pulmonary hypertension. Int J Mol Sci.

[188] Reynolds J.O., Quick A.P., Wang Q. (2016). Junctophilin-2 gene therapy rescues heart failure by normalizing RyR2-mediated Ca2+ release. Int J Cardiol.

[189] Prisco S.Z., Hartweck L.M., Kazmirczak F. (2024). Junctophilin-2 regulates mitochondrial metabolism. Circulation.

[190] Prins K.W., Asp M.L., Zhang H., Wang W., Metzger J.M. (2016). Microtubule-mediated misregulation of Junctophilin-2 underlies T-tubule disruptions and calcium mishandling in mdx mice. JACC Basic Transl Sci.

[191] Krasi G., Precone V., Paolacci S. (2019). Genetics and pharmacogenetics in the diagnosis and therapy of cardiovascular diseases. Acta Biomed.

[192] Strianese O., Rizzo F., Ciccarelli M. (2020). Precision and personalized medicine: how genomic approach improves the management of cardiovascular and neurodegenerative disease. Genes (Basel).

[193] Park J.F., Clark V.R., Banerjee S. (2021). Transcriptomic analysis of right ventricular remodeling in two rat models of pulmonary hypertension: identification and validation of epithelial-to-mesenchymal transition in human right ventricular failure. Circ Heart Fail.

[194] Zargarzadeh A., Javanshir E., Ghaffari A., Mosharkesh E., Anari B. (2023). Artificial intelligence in cardiovascular medicine: an updated review of the literature. J Cardiovasc Thorac Res.

[195] Biernacka E.K., Borowiec K., Franaszczyk M. (2021). Pathogenic variants in plakophilin-2 gene (PKP2) are associated with better survival in arrhythmogenic right ventricular cardiomyopathy. J Appl Genet.

[196] National Institutes of Health Open-label, dose escalation study of safety and preliminary efficacy of TN-401in adults with PKP2 mutation-associated ARVC (RIDGE-1). https://clinicaltrials.gov/study/NCT06228924.

[197] National Institutes of Health Gene Therapy for ACM due to a PKP2 pathogenic variant. https://www.clinicaltrials.gov/study/NCT06109181.

[198] Duong S.Q., Vaid A., My V.T.H. (2024). Quantitative prediction of right ventricular size and function from the ECG. J Am Heart Assoc.

[199] Balcioglu O., Ozgocmen C., Ozsahin D.U., Yagdi T. (2024). The role of artificial intelligence and machine learning in the prediction of right heart failure after left ventricular assist device implantation: a comprehensive review. Diagnostics (Basel).

[200] Qin Y., Qin X., Zhang J., Guo X. (2024). Artificial intelligence: the future for multimodality imaging of right ventricle. Int J Cardiol.

[201] Leiner T., Rueckert D., Suinesiaputra A. (2019). Machine learning in cardiovascular magnetic resonance: basic concepts and applications. J Cardiovasc Magn Reson.

[202] Ishikita A., McIntosh C., Hanneman K. (2023). Machine learning for prediction of adverse cardiovascular events in adults with repaired tetralogy of Fallot using clinical and cardiovascular magnetic resonance imaging variables. Circ Cardiovasc Imaging.

[203] Pezel T., Sanguineti F., Garot P. (2022). Machine-learning score using stress CMR for death prediction in patients with suspected or known CAD. JACC Cardiovasc Imaging.

[204] Fortuni F., Ciliberti G., De Chiara B. (2024). Advancements and applications of artificial intelligence in cardiovascular imaging: a comprehensive review. Eur Heart J Imaging Methods Pract.

